# Supplementary material for: Woven organic crystals
Source: Nat Commun. 2023 Nov 21;14:7582. doi: 10.1038/s41467-023-43084-7 (PMC10663483; doi:10.1038/s41467-023-43084-7)
Supplement: Supplementary file 1 — Supplementary Information [file 41467_2023_43084_MOESM1_ESM.pdf]

Supplementary Information

Woven Organic Crystals

Lan et al.

Content

1. Characterization **3**

2. Supplementary figures **4**

3. Supplementary tables **27**

4. Supplementary references **35**

1. Characterization.

The UV−vis absorption spectra were recorded with Shimadzu UV-2550 spectrophotometer. The emission spectra were recorded with Maya2000 Pro CCD spectrometer. The absolute fluorescence quantum yields were acquired on an Edinburgh FLS920 spectrometer combined with a calibrated integrating sphere. Environmental scanning electron microscopy photographs were obtained on FEI Quanta 450 environmental scanning electron microscope (ESEM) operated at 5 – 10 kV. Optical photographs of crystals were obtained by using a Canon camera or an optical Olympus BX61 microscope. The three-point bending and tensile tests were carried out using an Instron 5944 universal testing system with a capacity of 5 N Instron 2530 load cell. The high- and low-temperature environments were obtained by a Yamato DP23C vacuum drying oven and a cryogenic Dewar bottle containing liquid nitrogen, respectively. Differential scanning calorimetric (DSC) measurements were carried out on a TA DSC Q20 instrument.

2. Supplementary figures

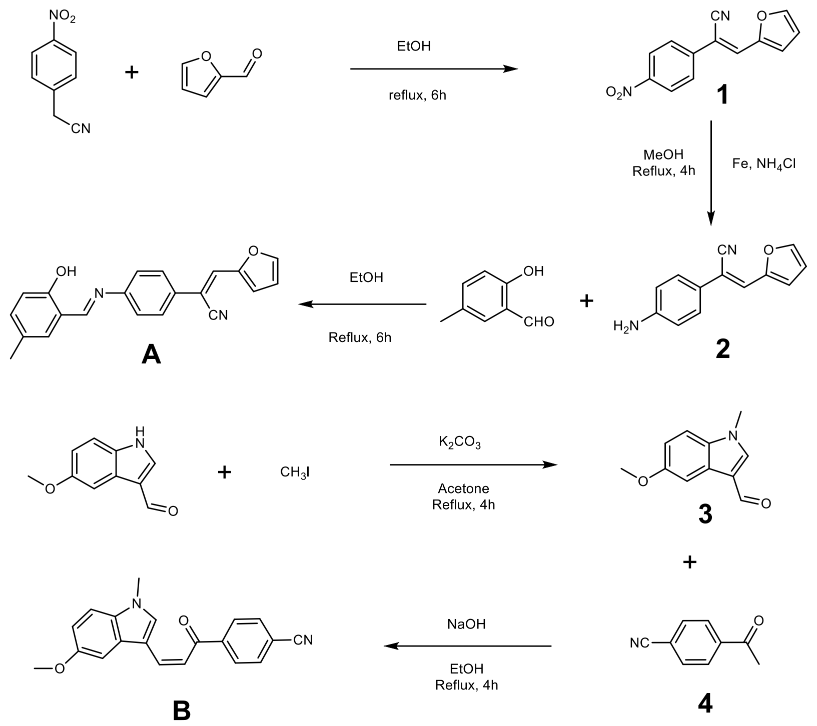

Supplementary Figure 1. The synthetic procedures for compounds **A** and **B**.

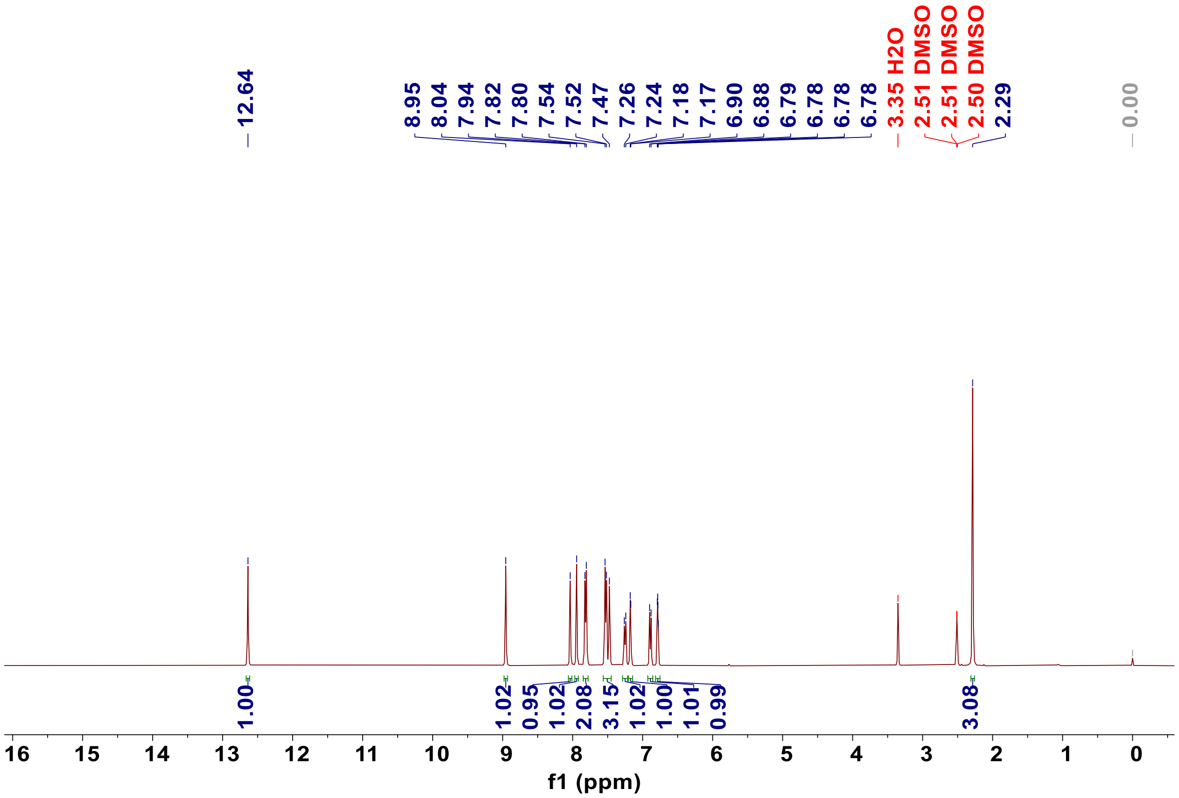

Supplementary Figure 2. ^{1}H NMR spectrum of compound **A** (DMSO-d_6, 400 MHz).

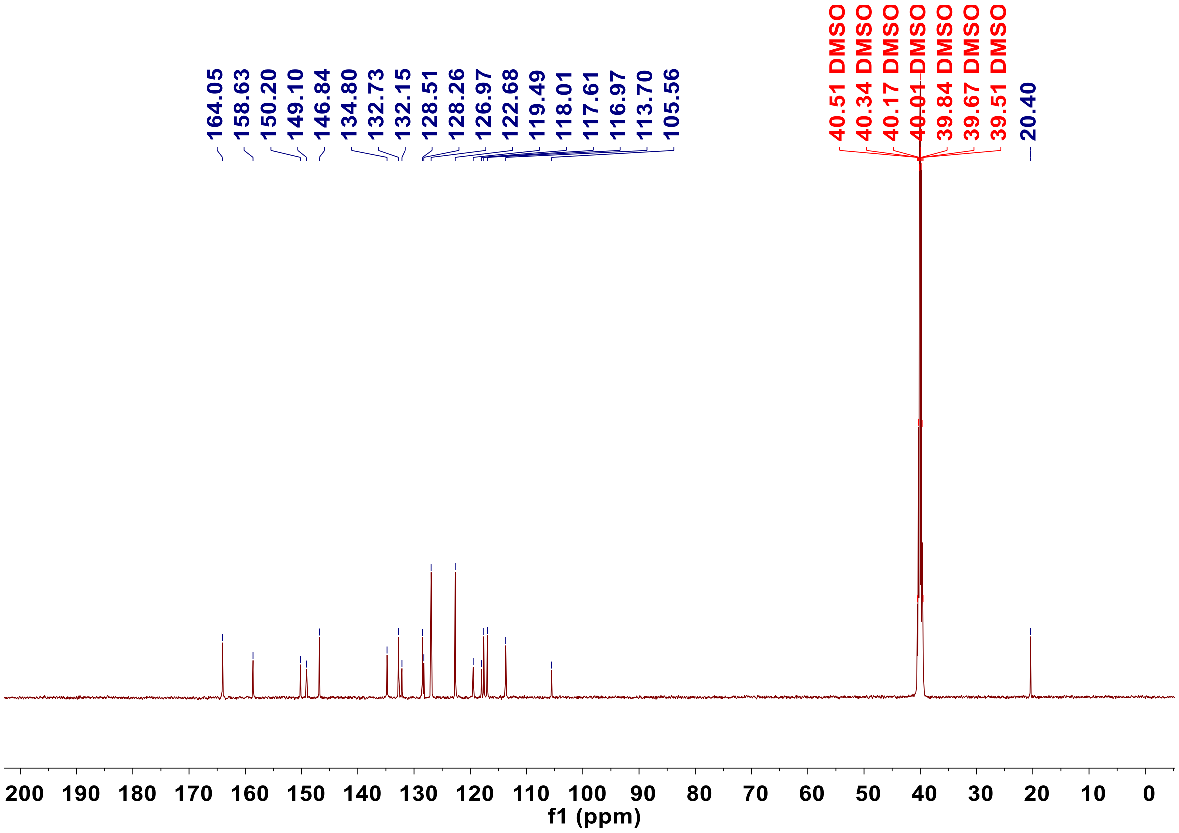

Supplementary Figure 3. $^{13}C\{^{1}H\}$ NMR spectrum of compound **A** (DMSO-d_6, 126 MHz).

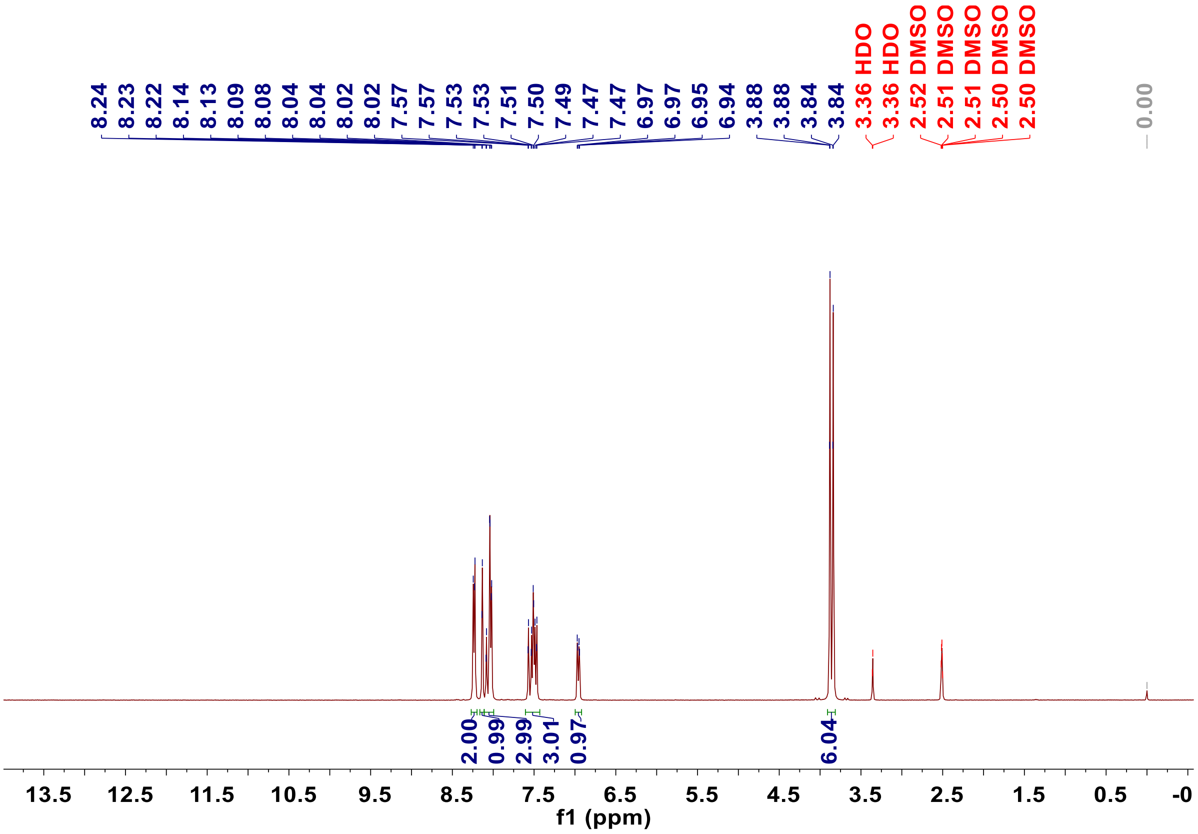

Supplementary Figure 4. ^{1}H NMR spectrum of compound **B** (DMSO-d_6, 400 MHz).

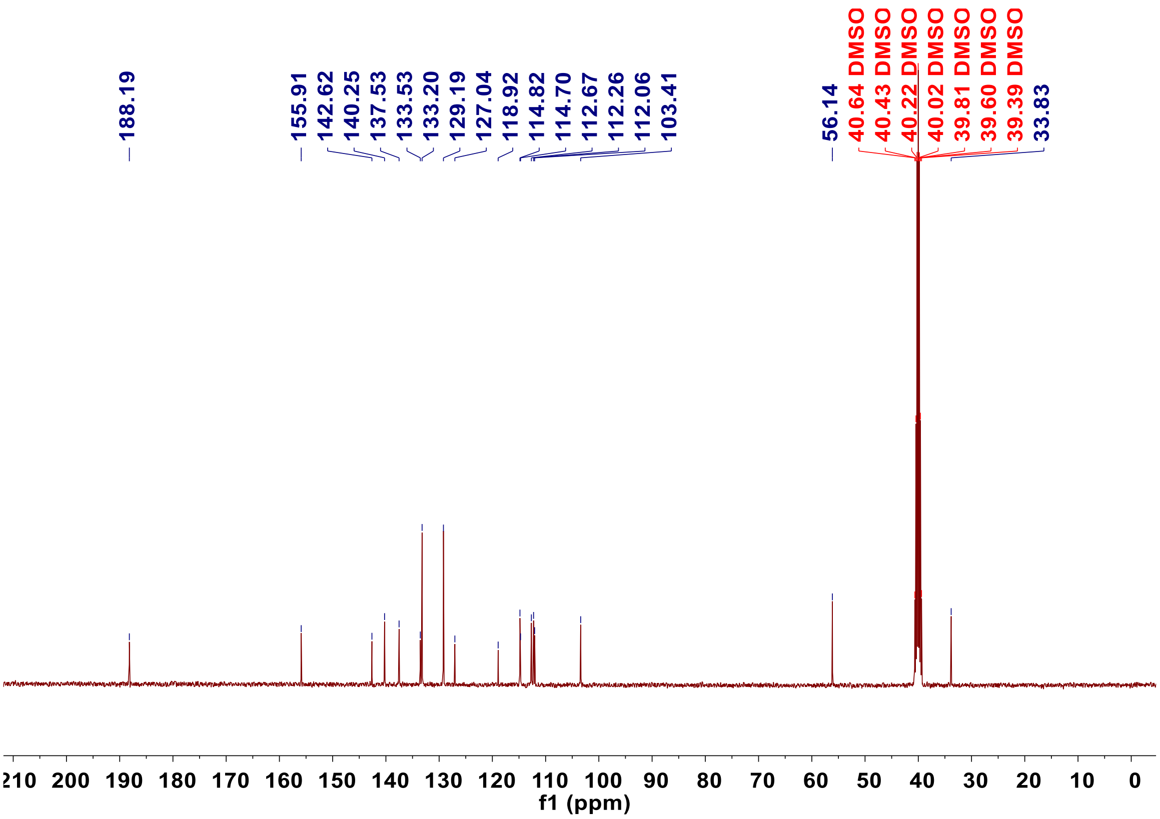

Supplementary Figure 5. $^{13}C\{^{1}H\}$ NMR spectrum of compound **B** (DMSO-d_6, 101 MHz).

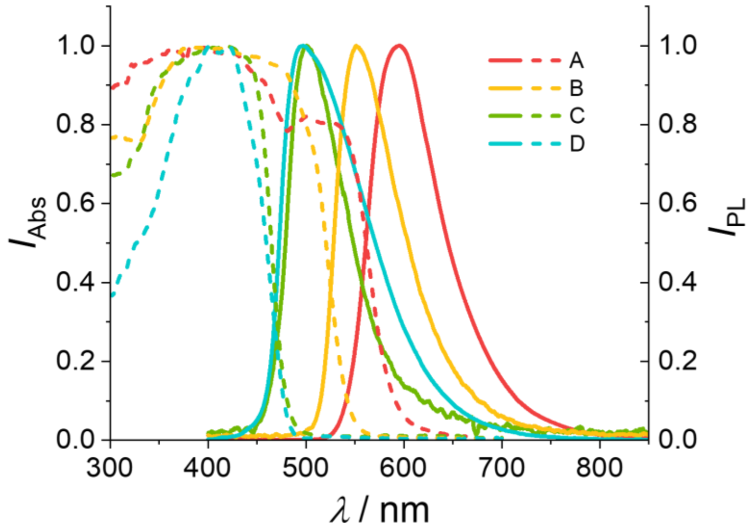

Supplementary Figure 6. Absorption (broken line) and fluorescence emission spectra (solid line) of crystals **A**–**D** recorded in single crystal.

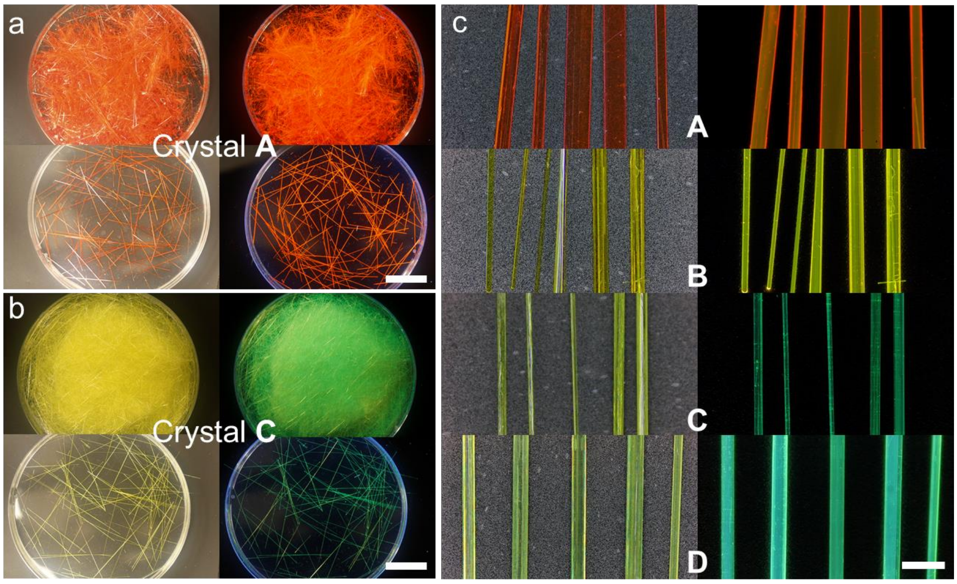

Supplementary Figure 7. (a, b) Photographs of the grown (top) and selected (bottom) crystals **A** and **C** in daylight (top) and under UV light (bottom). (c) Photomicrographs of crystals **A**–**D** in daylight (left) and under UV light (right). The scale bar in panels a and b is 2 cm, and in panel c is 500 µm.

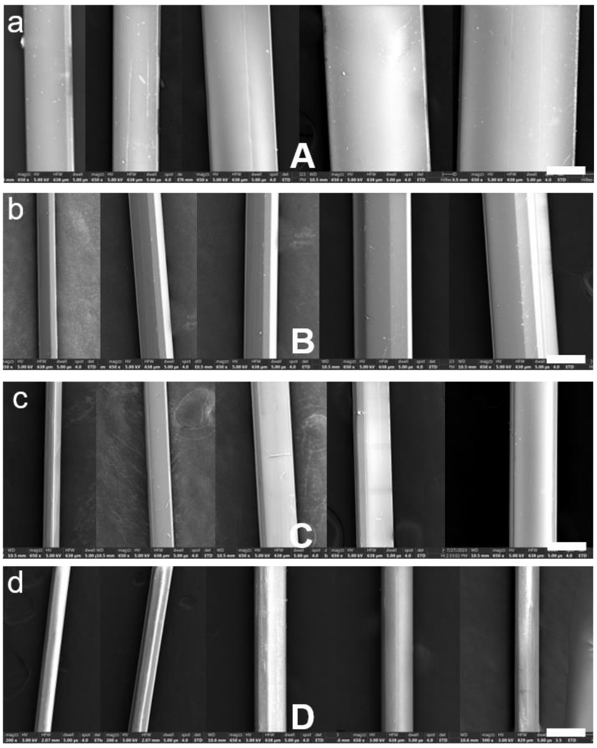

Supplementary Figure 8. Scanning electron micrographs showing surfaces of crystals **A** (a), **B** (b), **C** (c) and **D** (d). The scale bar in all panels is 100 µm.

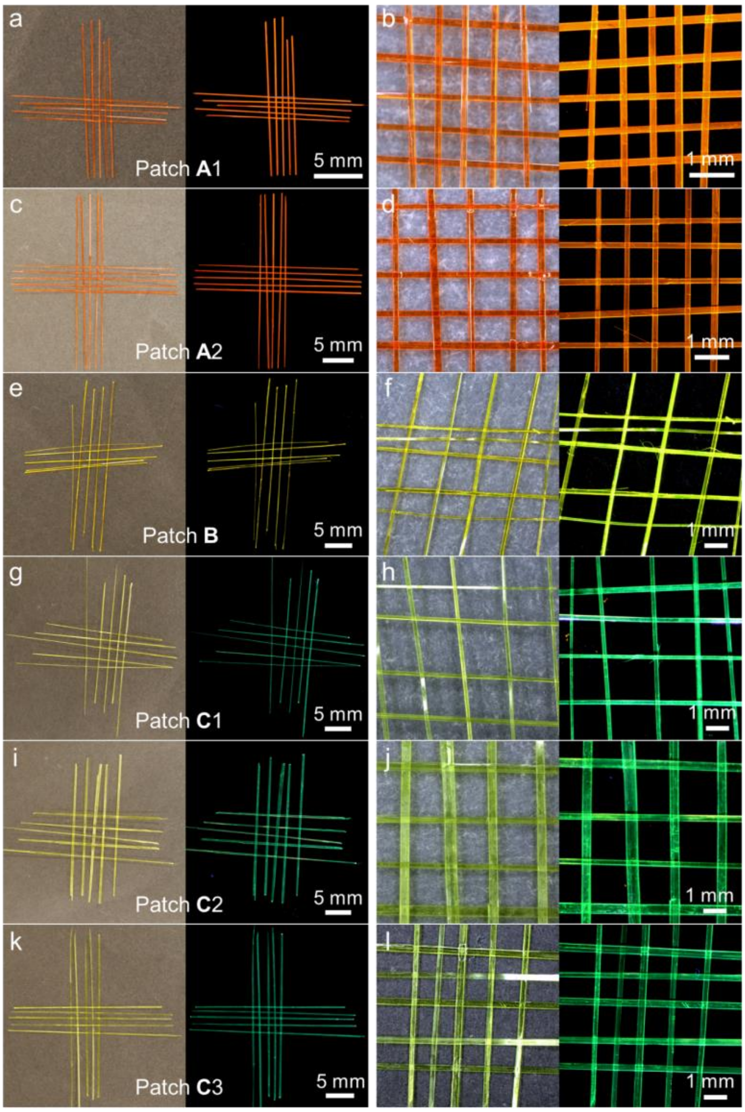

Supplementary Figure 9. Photographs and zoomed-in images of crystalline patches **A**1 (a, b), **A**2 (c, d), **B** (e, f), **C**1 (g, h), **C**2 (i, j) and **C**3 (k, l) recorded in daylight (left) and under UV light (right).

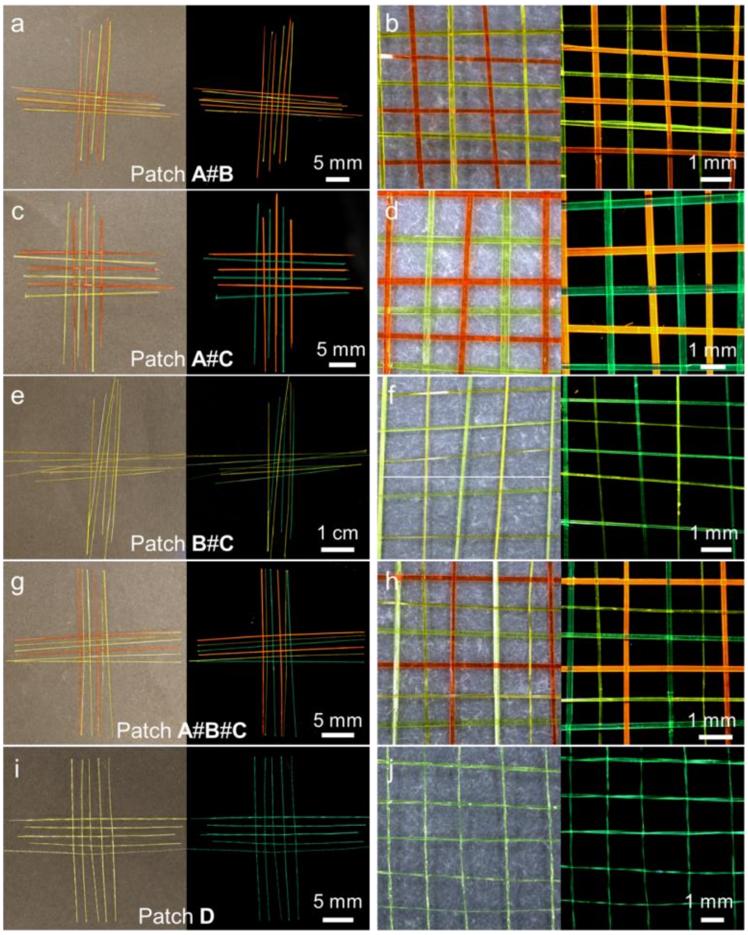

Supplementary Figure 10. Photographs and zoomed-in images of crystalline patches **A#B** (a, b), **A#C** (c, d), **B#C**(e, f), **A#B#C** (g, h), and twisted **D** (i, j) recorded in daylight (left) and under UV light (right).

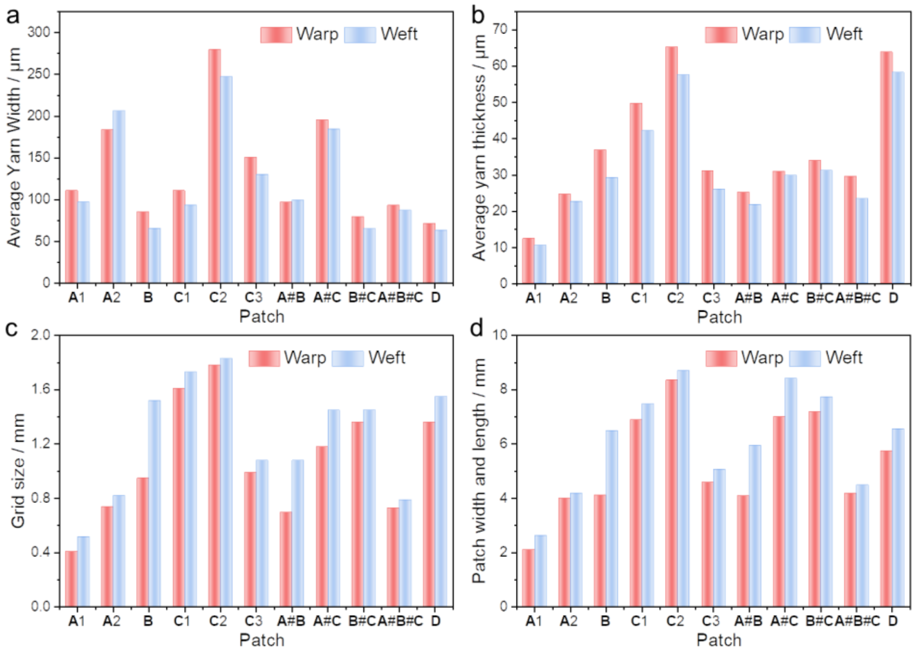

Supplementary Figure 11. Histograms showing the average crystal width (a), average crystal

thickness (b), average grid size (c) and fabric width and length (d) of eleven crystalline patches.

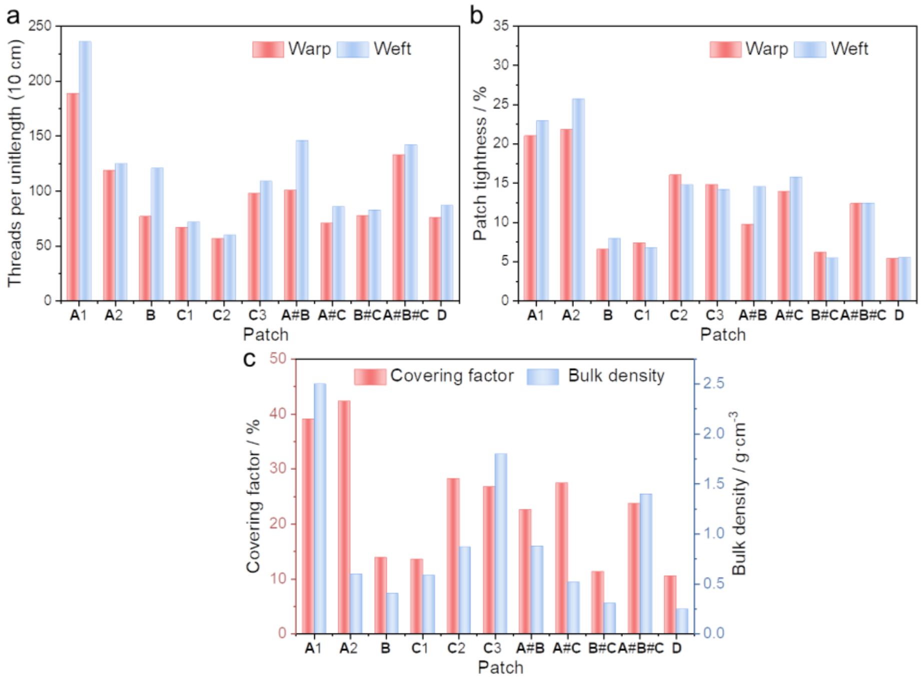

Supplementary Figure 12. Histograms showing the comparison of line density (a), fabric tightness (b), coverage factor and bulk density (c) of eleven crystalline patches.

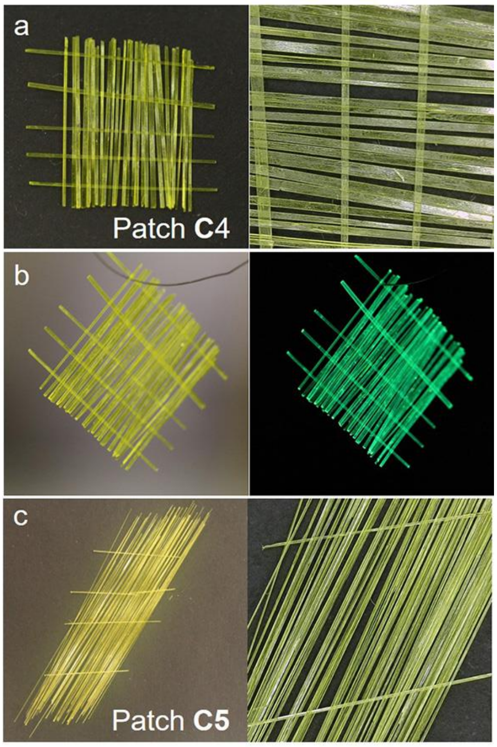

Supplementary Figure 13. (a) Photographs and zoomed-in images of the crystalline patch **C**4. (b) The patch suspended in air. (c) Photographs and zoomed-in images of the patch **C**5.

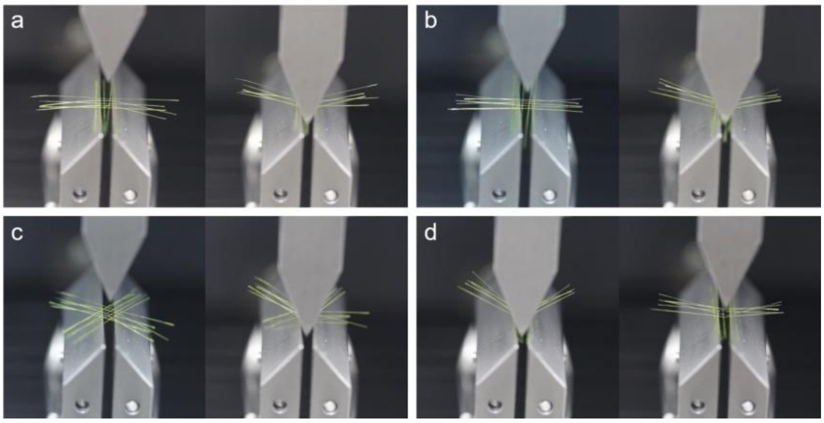

Supplementary Figure 14. Snapshots of the three-point bending tests of the crystalline patch **C**3. (a–c) Pressure is applied perpendicular to the weft (a), the warp (b), and in diagonal direction (c). (d) Three-point bending applied to the weft of **C**3 until the crystals were broken.

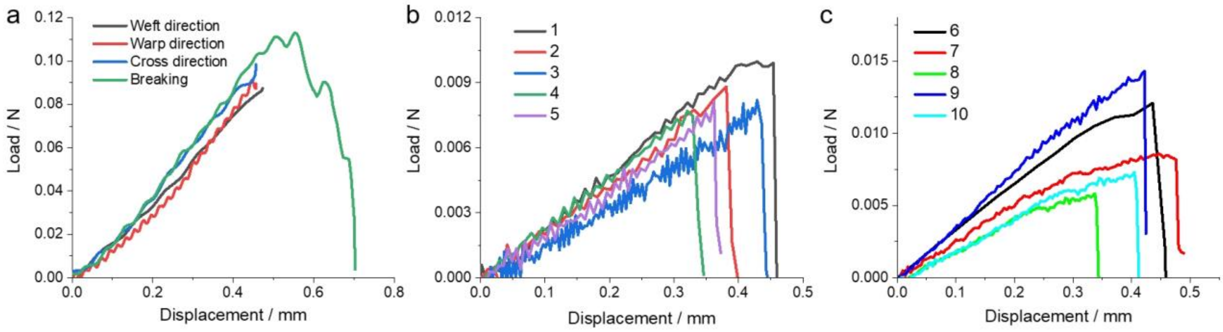

Supplementary Figure 15. Load–displacement curves obtained from the three-point bending tests of the patch **C**3 (a) and of the corresponding five weft crystals (b) and five warp crystals (c).

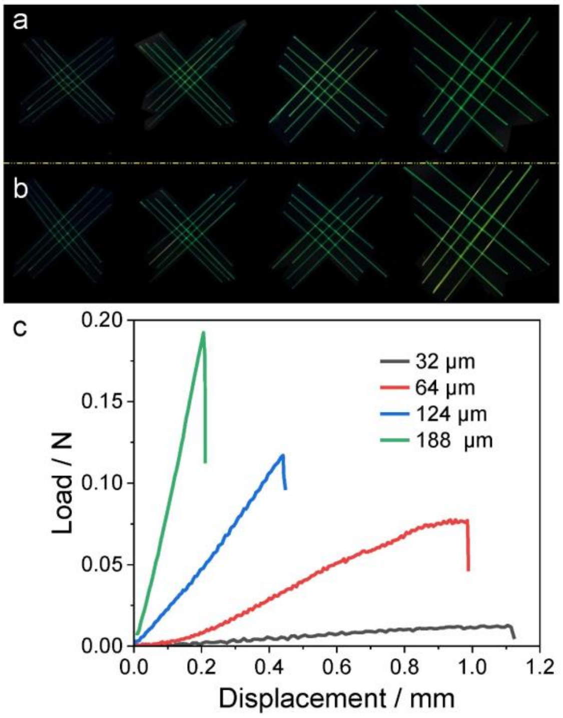

Supplementary Figure 16. (a, b) Fluorescence images of crystalline patches **C** of four different thicknesses (the average thickness of the crystals from left to right: 32, 64, 124, 188 μm) before (a) and after (b) the three-point bending experiments. (c) Load-displacement curves obtained from those crystalline patches **C**.

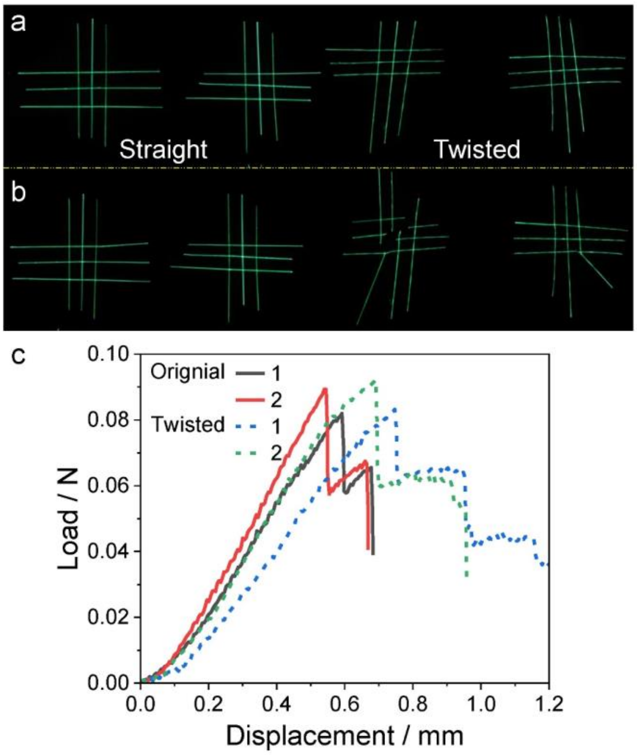

Supplementary Figure 17. (a, b) Fluorescence images of four crystalline patches **D** in straight and twisted states before (a) and after (b) the three-point bending experiments. (c) Load-displacement curves obtained from those crystalline patches **D**.

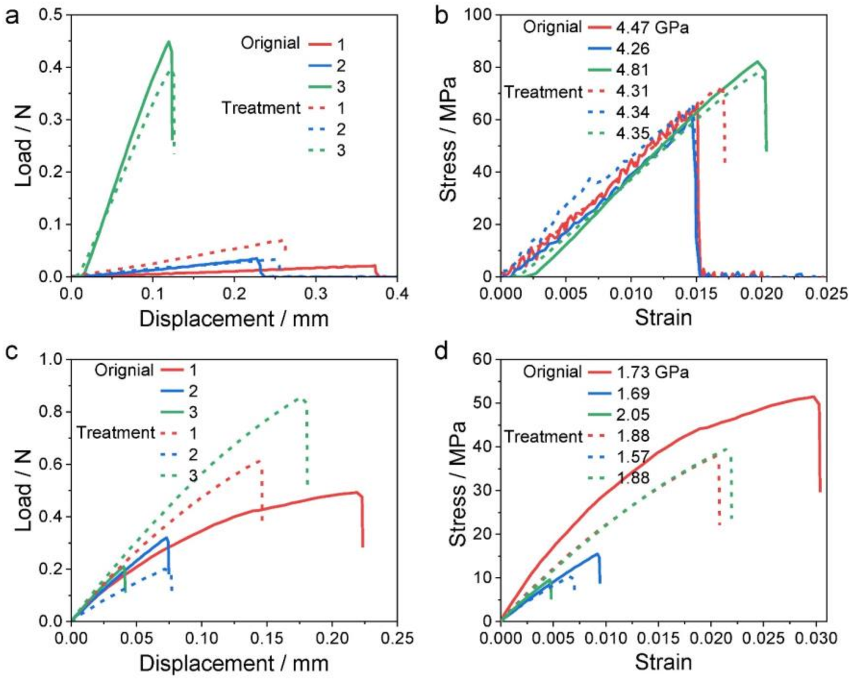

Supplementary Figure 18. (a, b) Load–displacement (a) and stress-strain (b) curves of crystals **C** before and after immersion in boiling water obtained from three-point bending tests. (c, d) Load–displacement (a) and stress-strain (b) curves of crystals **C** before and after immersion in boiling water obtained from tensile tests.

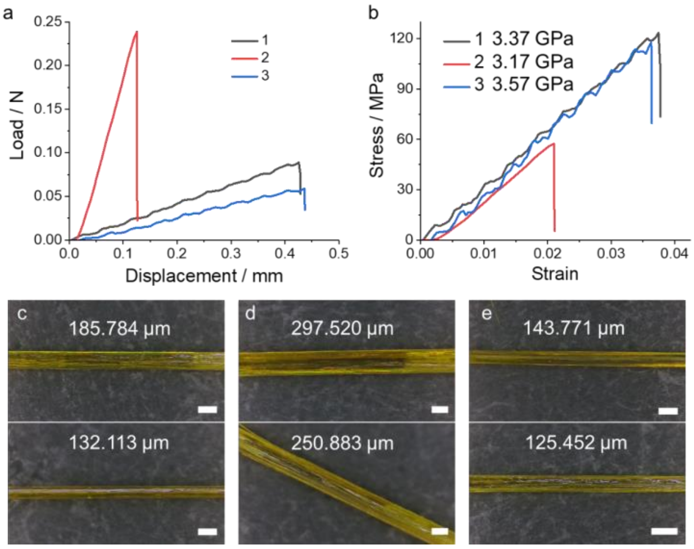

Supplementary Figure 19. (a, b) Load–displacement (a) and stress–strain curves (b) obtained from the three-point bending tests of crystal **B**. (c–e) Photographs of the width and thickness of three samples used in the three-point bending tests. The scale length in all panels is 200 µm.

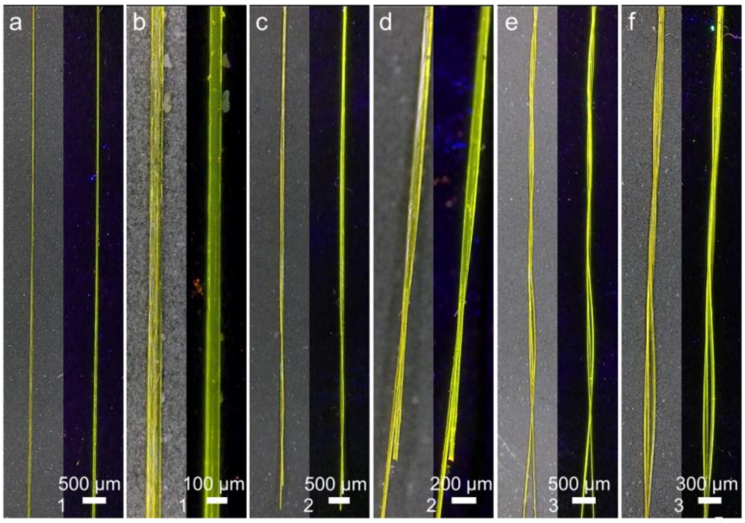

Supplementary Figure 20. Photographs of crystals **B** with single (a, b), double (c, d), and triple (e, f) strands recorded under daylight and under UV light.

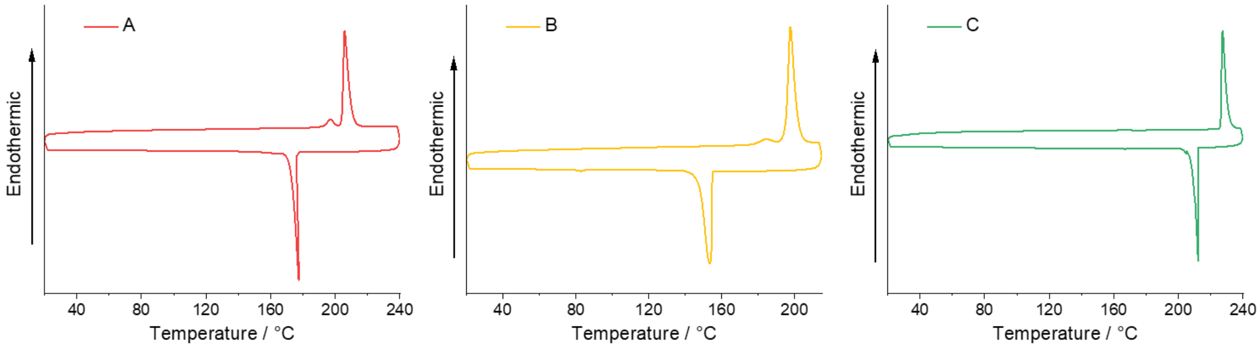

Supplementary Figure 21. Differential scanning calorimetric (DSC) curves of crystals **A**, **B** and **C**.

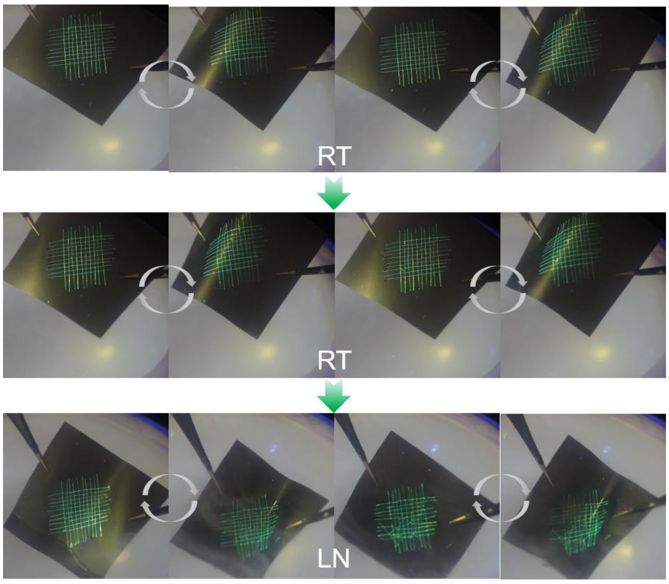

Supplementary Figure 22. Repeated bending of patch **C** at room temperature (RT) and immersed in liquid nitrogen (LN). The patch was attached to black paper which was bent.

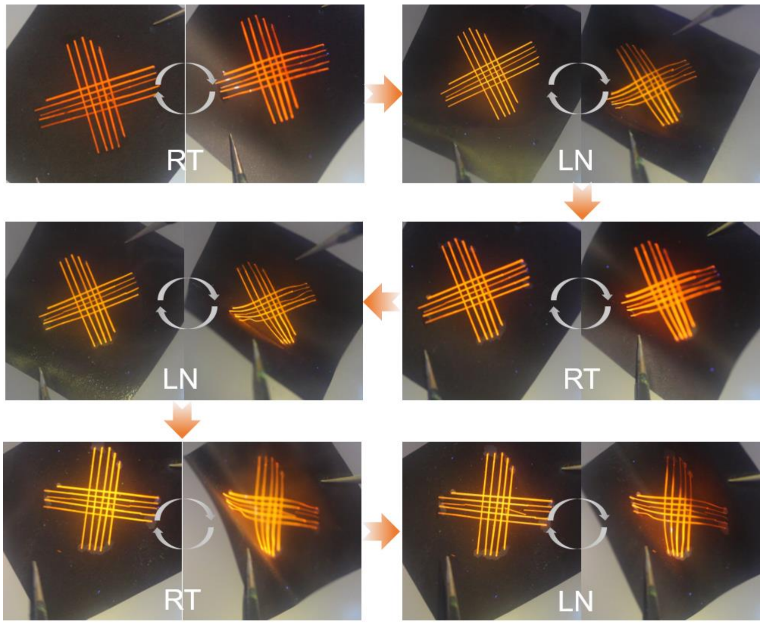

Supplementary Figure 23. Repeated bending of patch **A** at room temperature (RT) and immersed in liquid nitrogen (LN). The patch was attached to the black paper which was bent.

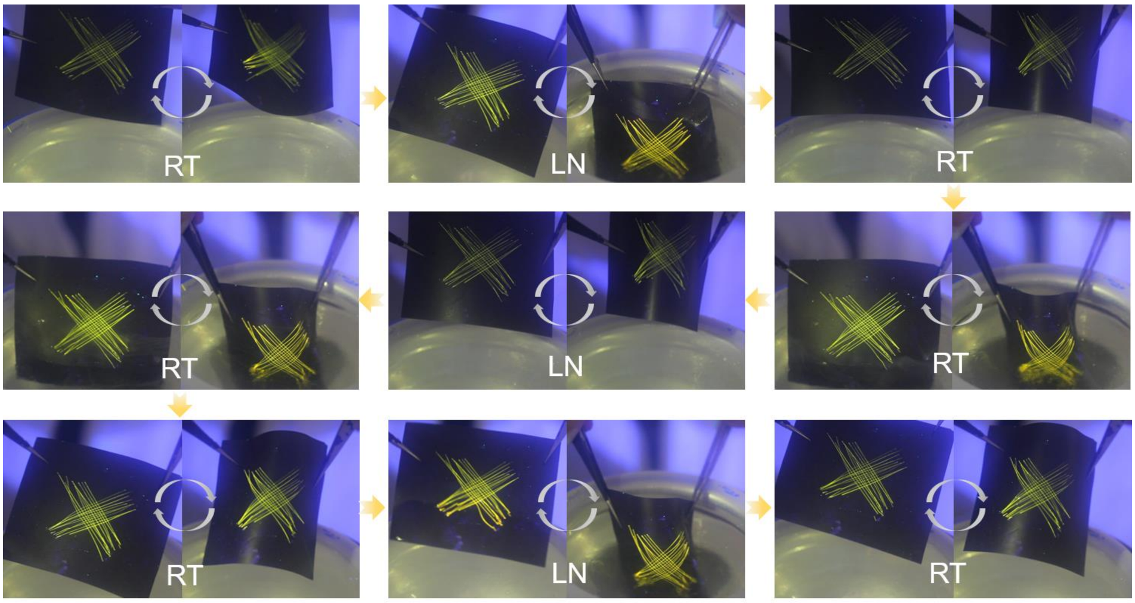

Supplementary Figure 24. Repeated bending patch **B** at room temperature (RT) and immersed in liquid nitrogen (LN). The patch was attached to the black paper and bent together.

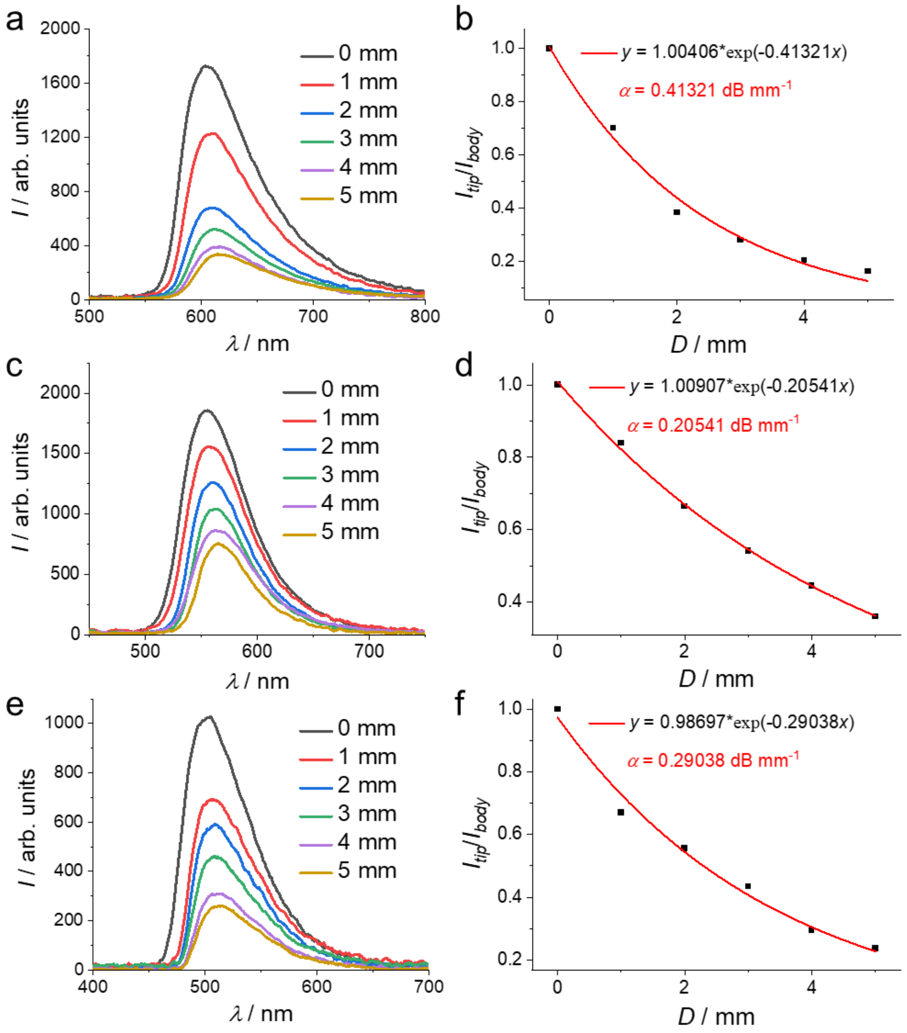

Supplementary Figure 25. Optical waveguiding properties of 5 mm crystals **A**–**C**. (a, c, e) Fluorescence spectra collected at one tip of the crystals **A** (a), **B** (c) and **C** (e) with different distances between the tip and the excitation site of the laser. (b, d, f) The $I_{\text{tip}}/I_{\text{body}}$ decays of crystals **A** (b), **B** (d) and **C** (f). The optical loss coefficients (α) were determined by single-exponential fitting of the function $I_{\text{tip}}/I_{\text{body}} = A\exp(-\alpha D)$, in which I_{tip} and I_{body} are the fluorescence intensities of outcoupled and incidence light respectively, A is the optical loss coefficient, and D is the distance between the excited site and the tip of crystals for collecting emission.

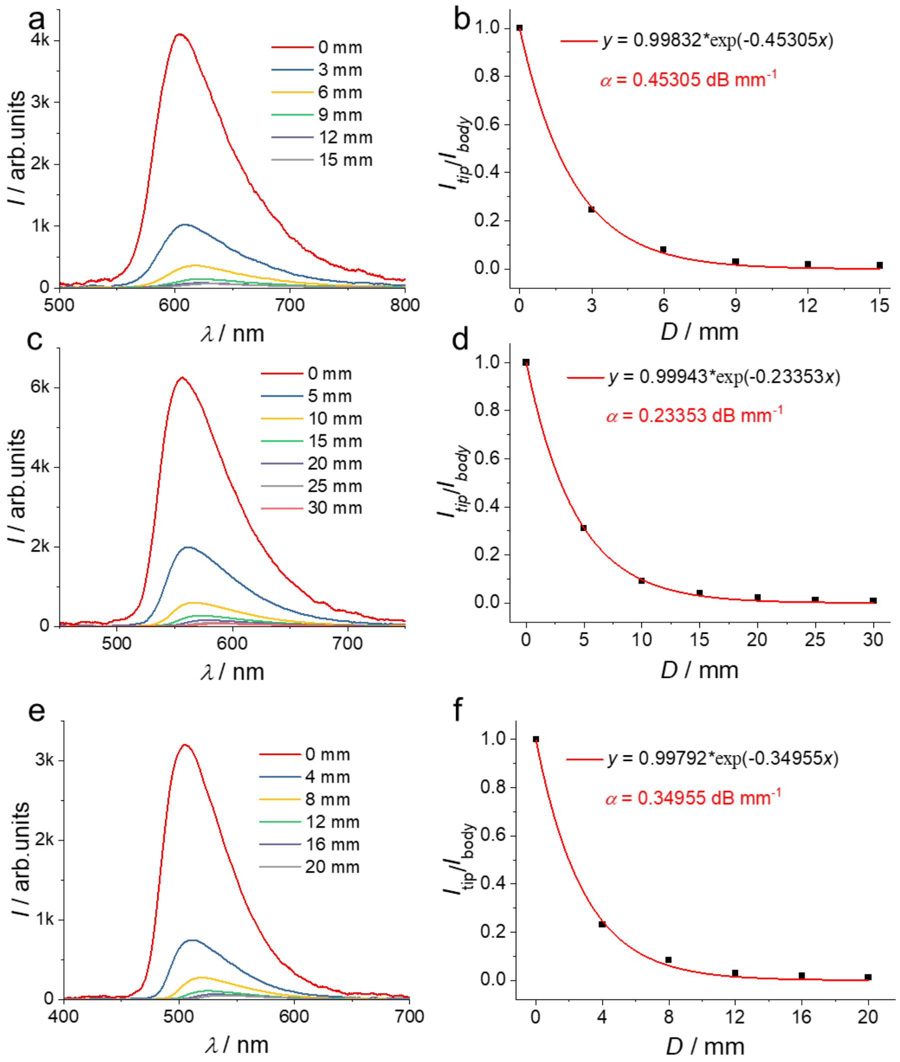

Supplementary Figure 26. Optical waveguiding properties of long size crystals **A**–**C**. (a, c, e) Fluorescence spectra collected at one tip of the crystals **A** (a), **B** (c) and **C** (e) with different distances between the tip and the excitation site of the laser. (b, d, f) The I_{tip}/I_{body} decays of crystals **A** (b), **B** (d) and **C** (f). The optical loss coefficients (α) were determined by single-exponential fitting of the function $I_{tip}/I_{body} = A\exp(-\alpha D)$, in which I_{tip} and I_{body} are the fluorescence intensities of outcoupled and incidence light respectively, A is the optical loss coefficient, and D is the distance between the excited site and the tip of crystals for collecting emission.

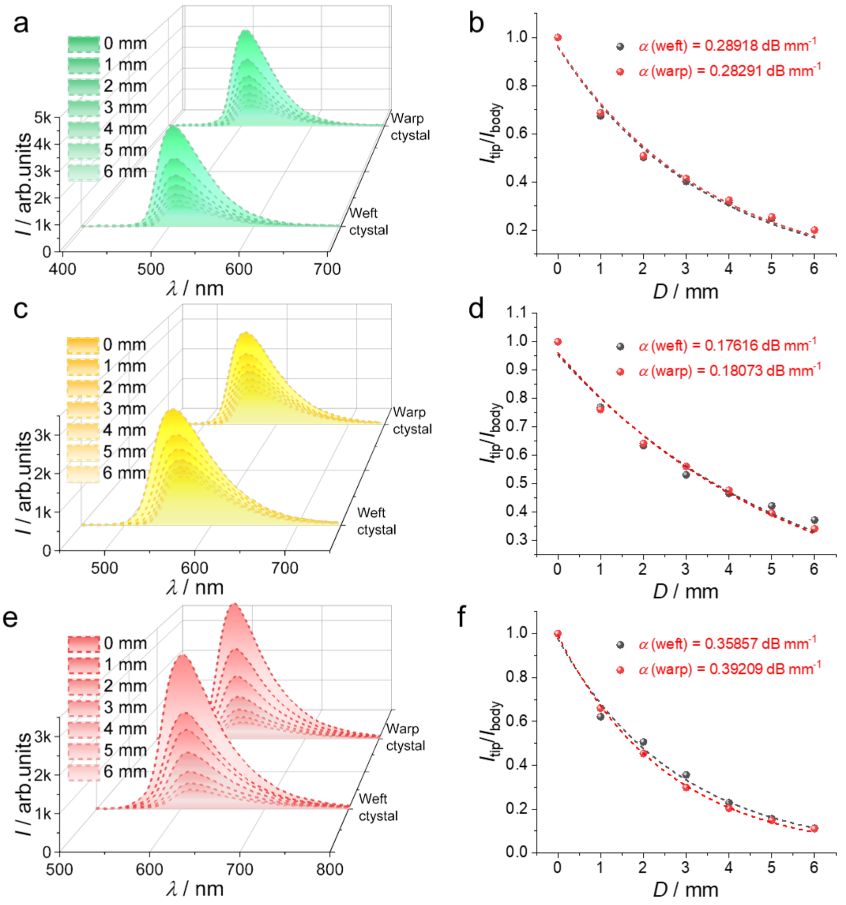

Supplementary Figure 27. Optical waveguiding properties of weft and warp crystals **A**–**C** of the patch **A#B#C**. (a, c, e) Fluorescence spectra collected at one tip of the weft and warp crystals **A** (a), **B** (c) and **C** (e) with different distances between the tip and the excitation site of the laser. (b, d, f) The I_{tip}/I_{body} decays of the weft and warp crystals **A** (b), **B** (d) and **C** (f). The optical loss coefficients (α) were determined by single-exponential fitting of the function $I_{tip}/I_{body} = A\exp(-\alpha D)$, in which I_{tip} and I_{body} are the fluorescence intensities of outcoupled and incidence light respectively, A is the optical loss coefficient, and D is the distance between the excited site and the tip of crystals for collecting emission.

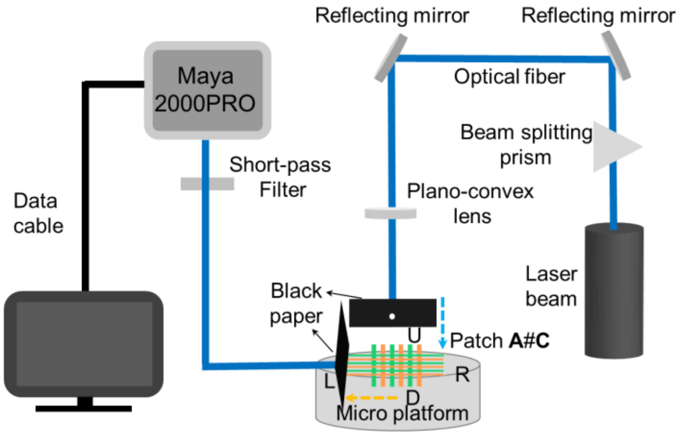

Supplementary Figure 28. Schematic diagram of the optical waveguide testing setup used to study the optical transmission of the crystalline patches. The blue dashed line indicates the laser transmission direction, and the yellow dashed line indicates light signal transmission direction of the excited crystals.

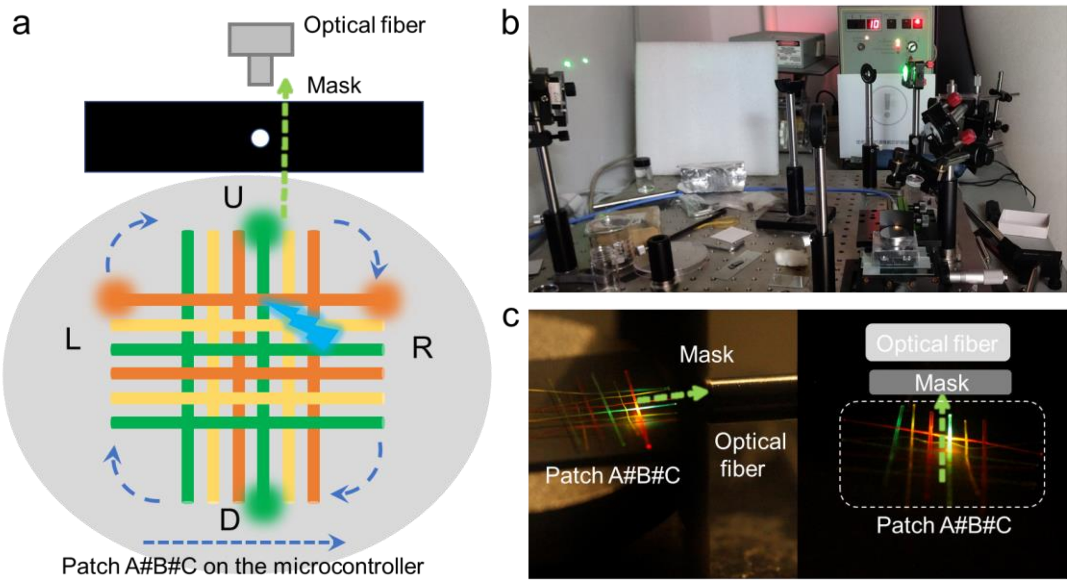

Supplementary Figure 29. (a, b) The Schematic (a) and set-up (b) for a crystalline patch used to collect the fluorescence spectra. (c) Optical waveguides of patch **A#B#C** observed from side (left) and front directions (right). The blue arrow represents a 355 nm laser. The green dotted arrows represent the direction of receiving the optical signal. The dashed blue arrows represent the direction in which the micro control patches move.

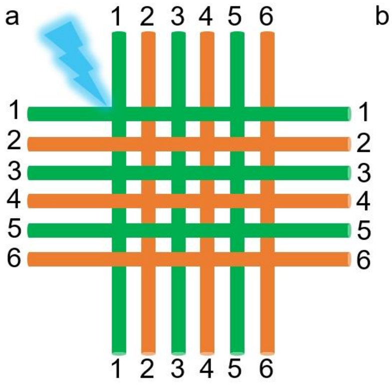

b

Wefts \ Warps	1	2	3	4	5	6
1	(0000)	(0101)	(0000)	(0101)	(0000)	(0101)
2	(1010)	(1111)	(1010)	(1111)	(1010)	(1111)
3	(0000)	(0101)	(0000)	(0101)	(0000)	(0101)
4	(1010)	(1111)	(1010)	(1111)	(1010)	(1111)
5	(0000)	(0101)	(0000)	(0101)	(0000)	(0101)
6	(1010)	(1111)	(1010)	(1111)	(1010)	(1111)

Supplementary Figure 30. (a) Schematic of a patch **A#C** with warp and weft crystals numbered 1–6. (b) All 36 output signals of the optical waveguide array based on **A#C** as different cross-nodes were excited by a 355 nm laser.

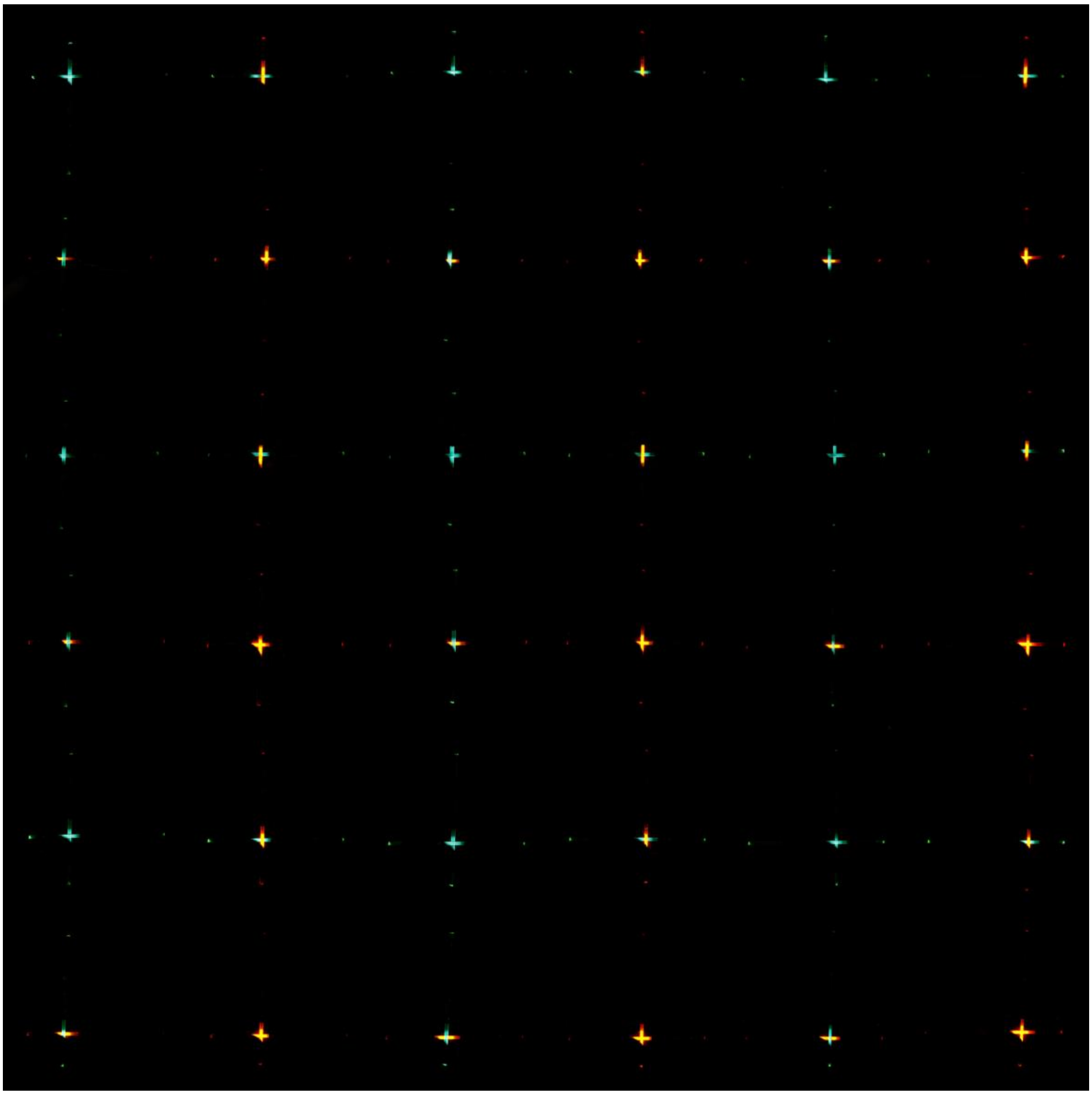

Supplementary Figure 31. Fluorescence images of the optical waveguides array of a patch **A#C** obtained by exciting **A#C** at three different positions including 36 cross-nodes.

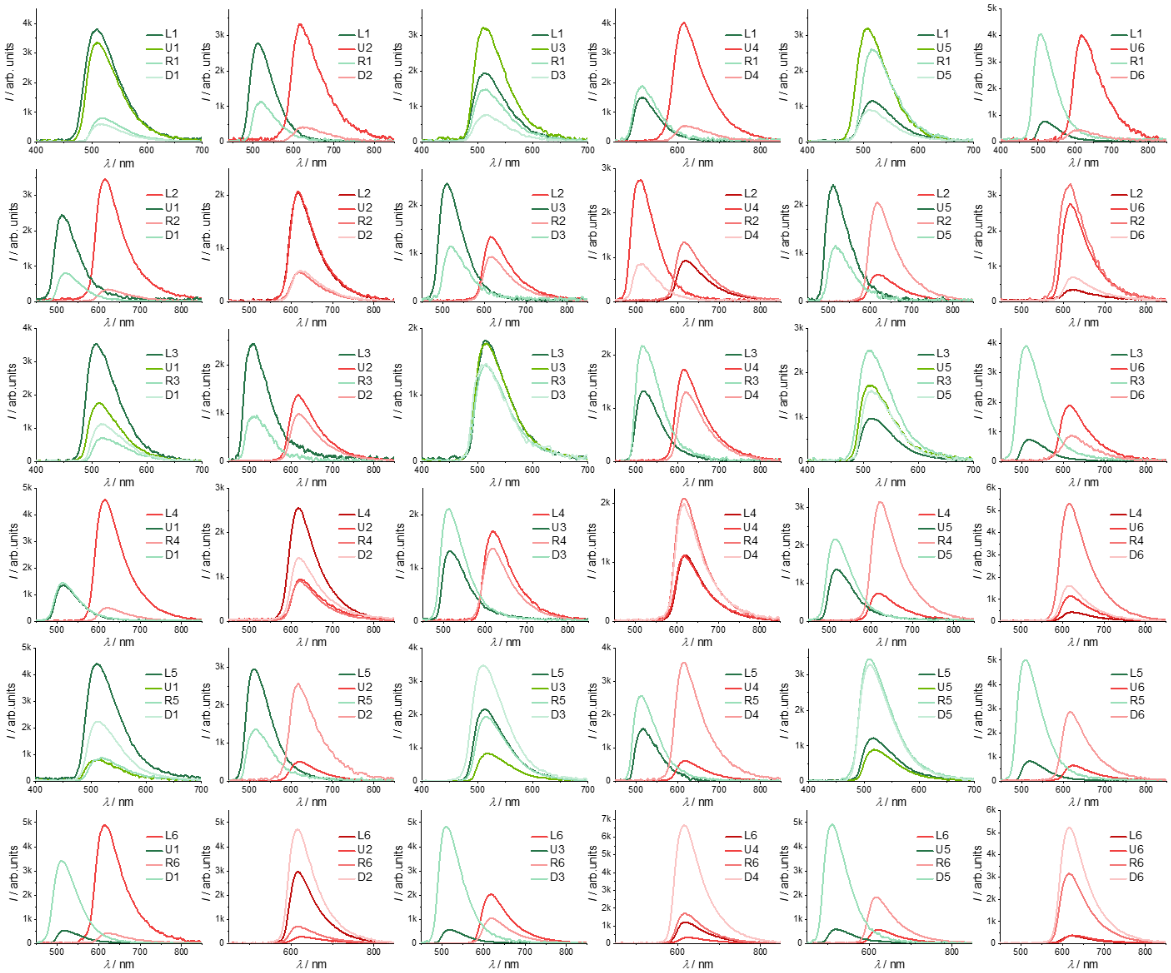

Supplementary Figure 32. Emission spectra collected at four ends of the patch **A#C** at various excitation positions including 36 cross-nodes.

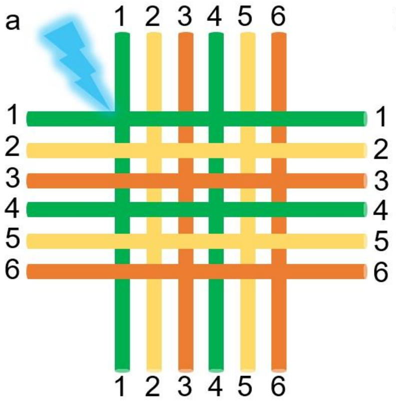

b

Wefts \ Warps	1	2	3	4	5	6
1	(0000)	(0101)	(0202)	(0000)	(0101)	(0202)
2	(1010)	(1111)	(1212)	(1010)	(1111)	(1212)
3	(2020)	(2121)	(2222)	(2020)	(2121)	(2222)
4	(0000)	(0101)	(0202)	(0000)	(0101)	(0202)
5	(1010)	(1111)	(1212)	(1010)	(1111)	(1212)
6	(2020)	(2121)	(2222)	(2020)	(2121)	(2222)

Supplementary Figure 33. (a) Schematic of a patch **A#B#C** with warp and weft crystals numbered 1–6. (b) All 36 output signals of the optical waveguide array based on **A#B#C** as different cross-nodes were excited by a 355 nm laser.

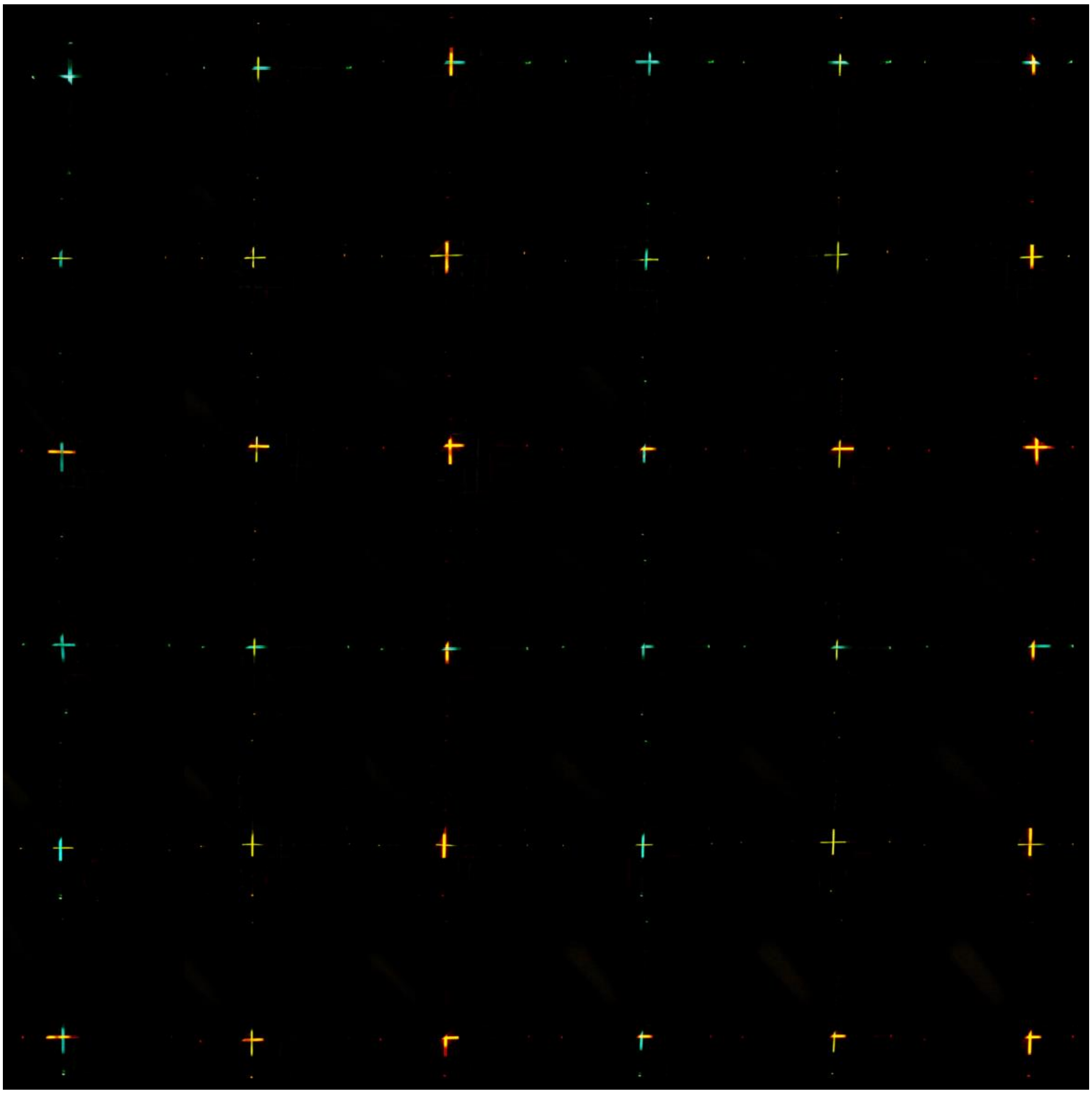

Supplementary Figure 34. Fluorescence images of the optical waveguides array of the patch **A#B#C** obtained by exciting the patch at three different positions including 36 cross-nodes.

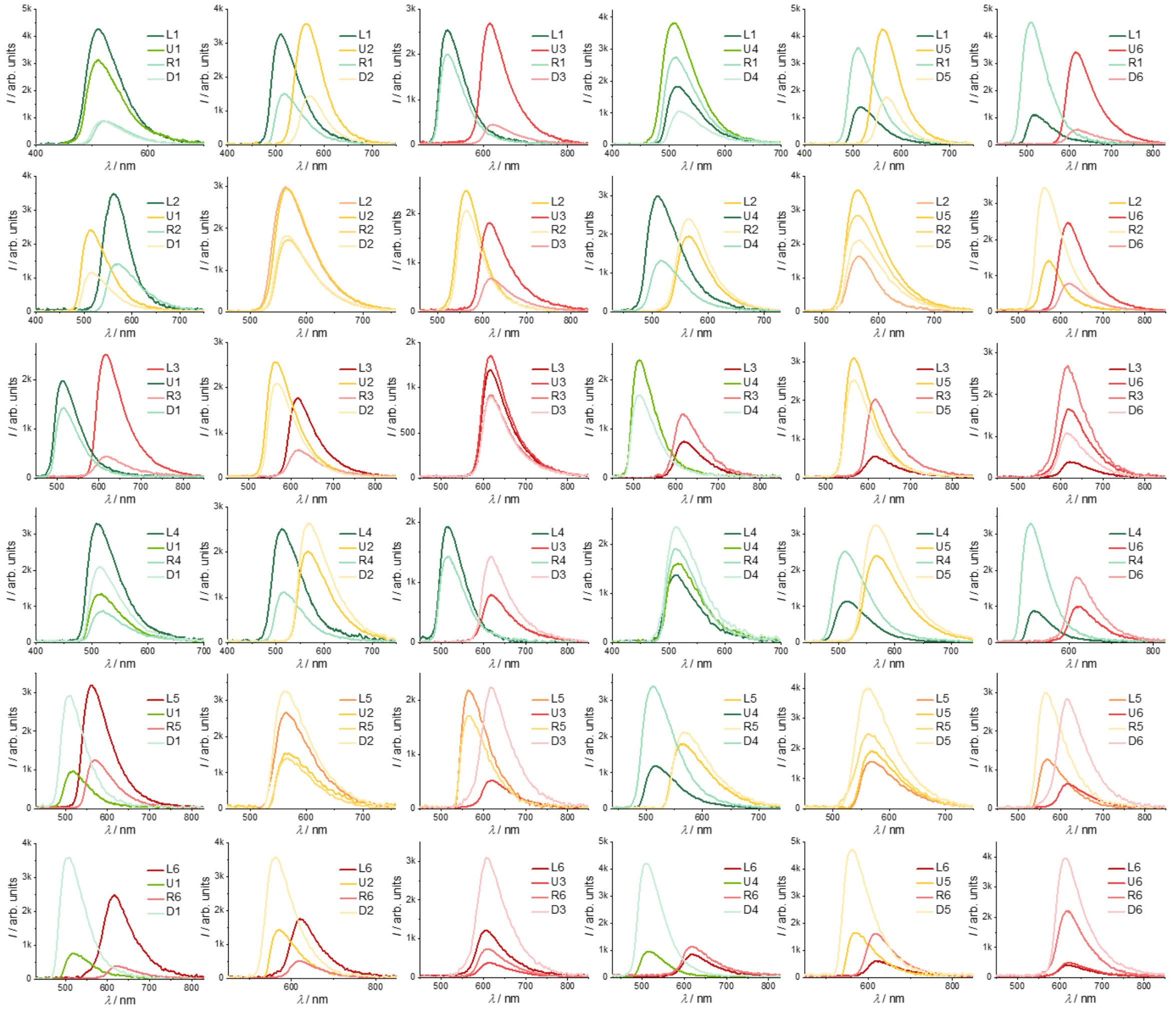

Supplementary Figure 35. Emission spectra collected at four ends of the patch **A#B#C** at various excitation positions including 36 cross-nodes.

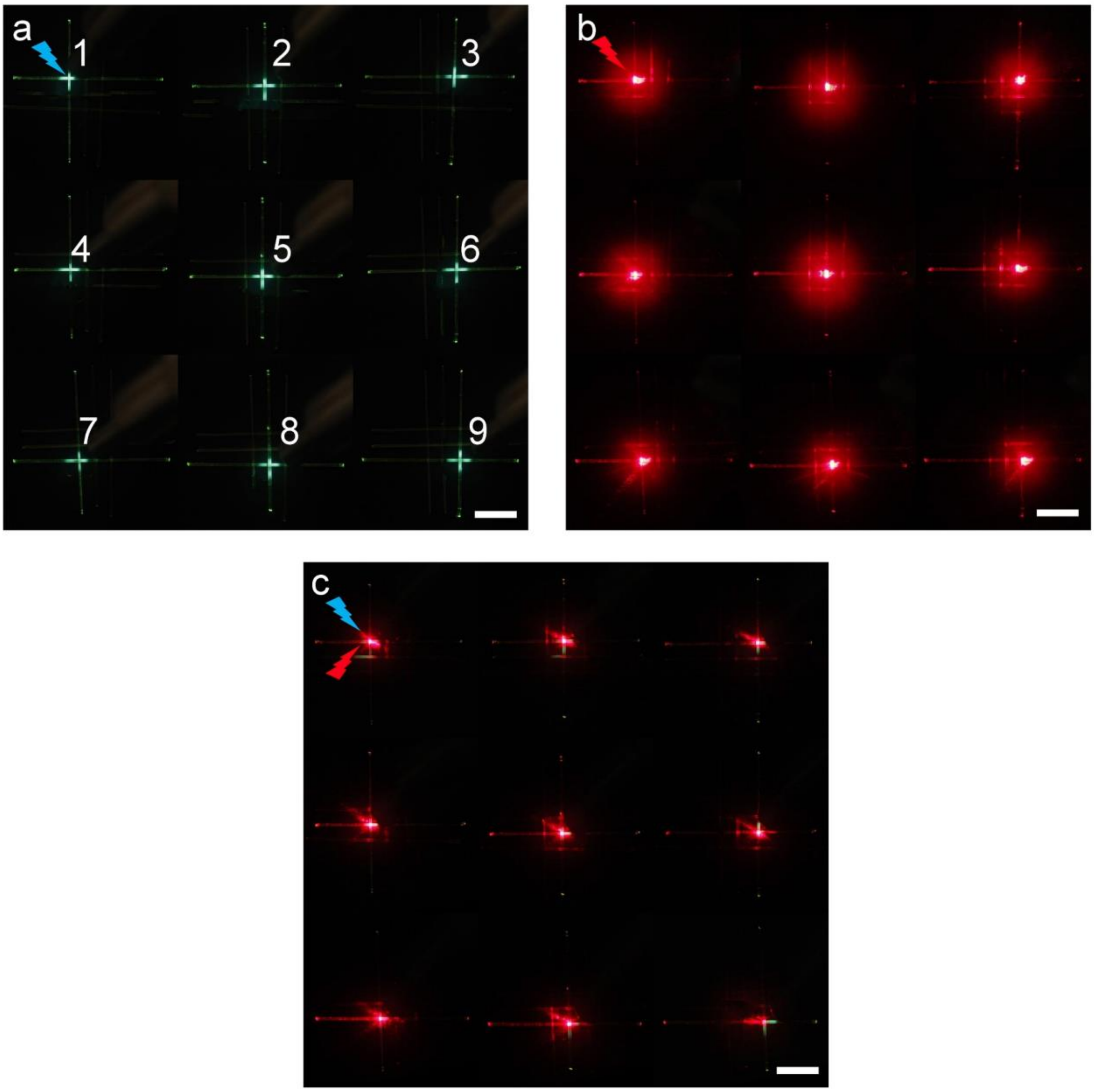

Supplementary Figure 36. Fluorescence images of the mode-1 obtained by exciting the patch at three different positions including 9 cross-nodes with 355 nm (a), 654 nm (b), and 355 nm combined with 654 nm lasers (c). The scale length in all panels is 4 mm.

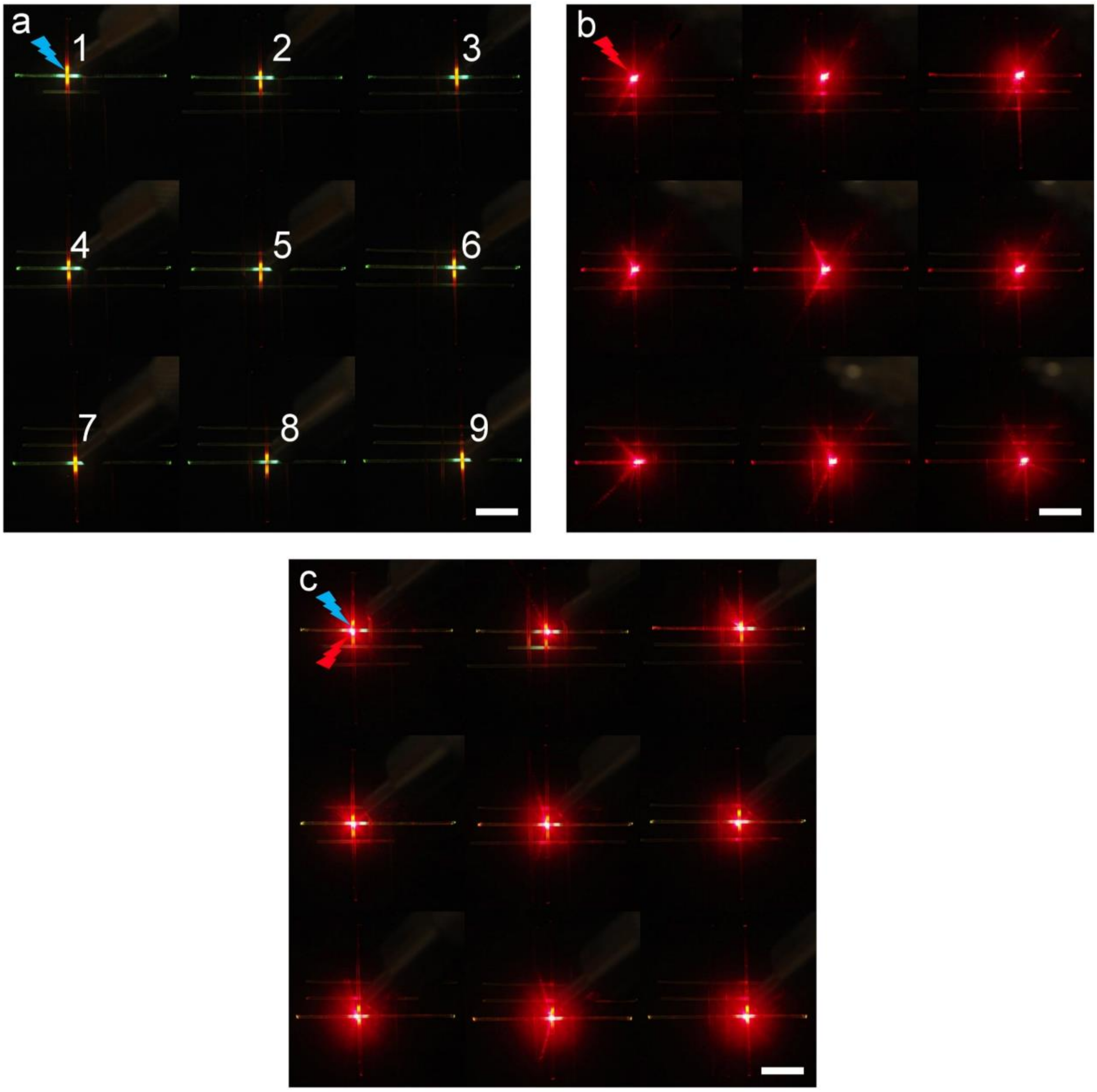

Supplementary Figure 37. Fluorescence images of mode-2 obtained by exciting the patch at three different positions including 9 cross-nodes with 355 nm (a), 654 nm (b), and 355 nm combined with 654 nm lasers (c). The scale length in all panels is 4 mm.

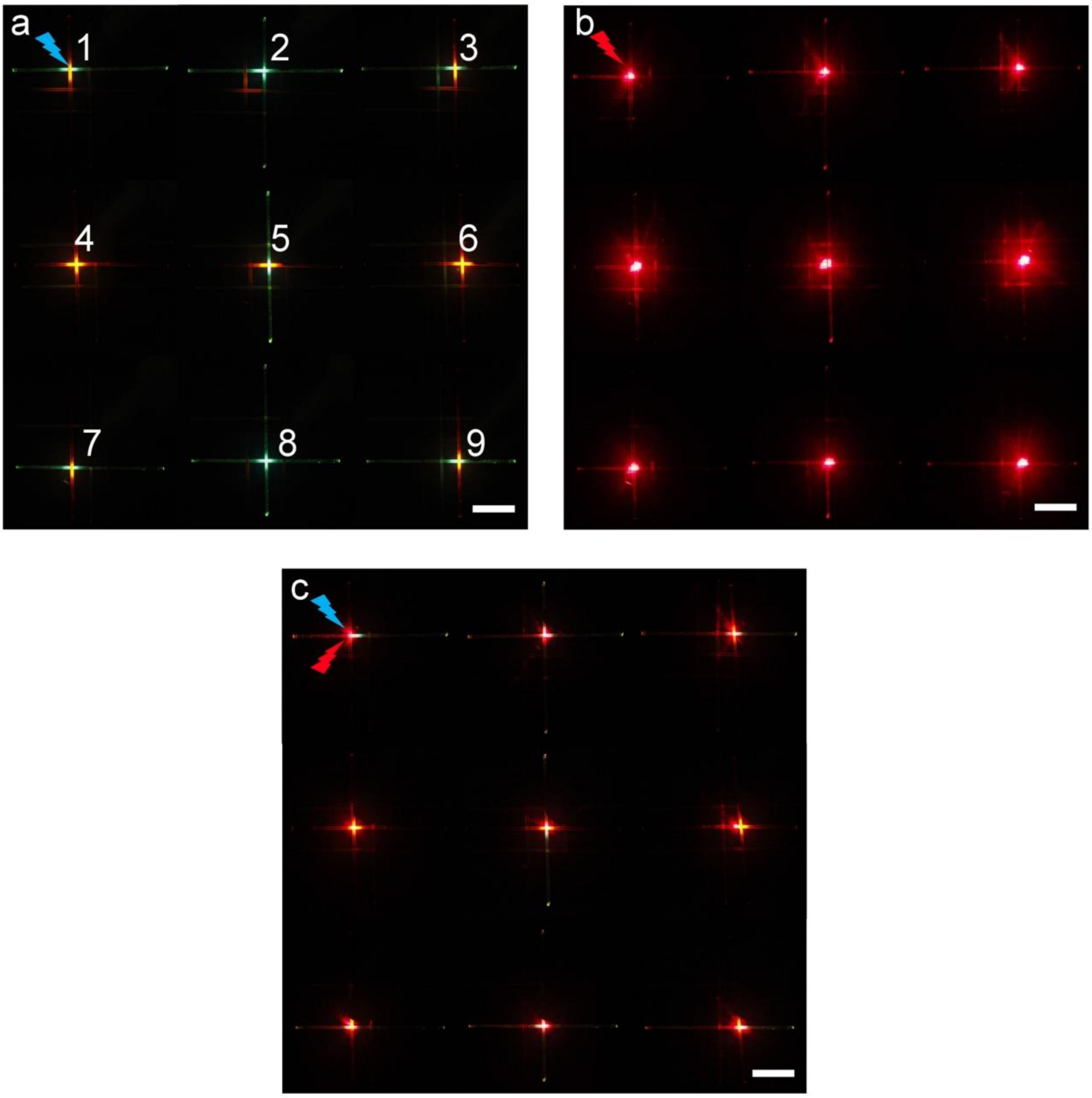

Supplementary Figure 38. Fluorescence images of the mode-3 obtained by exciting the patch at three different positions including 9 cross-nodes with 355 nm (a), 654 nm (b), and 355 nm combined with 654 nm lasers (c). The scale length in all panels is 4 mm.

3. Supplementary tables

Supplementary Table 1. Emission peak maxima (λ_{em}) and quantum yields (Φ) of crystals **A**–**D** at two sizes.

Crystal	length	λ_{em} (nm)	Φ (%)
**A**	< 1 mm	595	17.33
	~ 1 cm		17.07
**B**	< 1 mm	554	3.42
	~ 1 cm		3.24
**C**	< 1 mm	501	15.67
	~ 1 cm		14.99
**D**	< 1 mm	500	39.18
	~ 1 cm		37.50

Supplementary Table 2. Crystal data and structural refinement for the crystals **C** before and before and after immersion in boiling water.

Formula	$C_{14}H_8Br_2$ (Original)	$C_{14}H_8Br_2$ (Boiling water treatment)
Formula Weight	336.02	336.02
Temperature / K	273.15	273.15
Crystal System	triclinic	triclinic
Space Group	$P\bar{1}$	$P\bar{1}$
a / Å	4.0689(3)	4.0686(3)
b / Å	8.9080(7)	8.9081(7)
c / Å	16.1505(12)	16.1506(12)
α / °	99.082(3)	99.099(3)
β / °	96.916(3)	96.916(3)
γ / °	100.402(3)	100.416(3)
Volume / Å^3	561.83(7)	561.73(8)
Z	2	2
$\rho_{calc.}$ / g cm^{-3}	1.986	1.987
μ / mm^{-1}	7.179	7.180
F(000)	324.0	324.0
Size / mm^3	0.20 × 0.20 × 0.20	0.20 × 0.20 × 0.20
Radiation type	MoK$_\alpha$ (λ = 0.71073)	MoK$_\alpha$ (λ = 0.71073)
Θ_{min} / °	5.17	5.17
Θ_{max} / °	55.092	55.112
Index ranges	$-5 \le h \le 5, -11 \le k \le 11, -20 \le l \le 20$	$-5 \le h \le 5, -11 \le k \le 11, -20 \le l \le 20$
Reflections collected	22484	24193
Independent reflections	2596 [R_{int} = 0.0329, R_{sigma} = 0.0158]	2596 [R_{int} = 0.0316, R_{sigma} = 0.0147]

Formula	$C_{14}H_8Br_2$ (Original)	$C_{14}H_8Br_2$ (Boiling water treatment)
Data/restraints/parameters	2596 / 0 / 145	2599 / 0 / 145
Goodness-of-fit on F^2	1.224	1.189
Final R indexes [$I >= 2\sigma (I)$]	R_1 = 0.0486, wR_2 = 0.1210	R_1 = 0.0400, wR_2 = 0.1029
Final R indexes [all data]	R_1 = 0.0539, wR_2 = 0.1262	R_1 = 0.0446, wR_2 = 0.1068
Largest diff. peak / hole / e Å^{-3}	0.59 / –1.97	0.51 / –1.46

Supplementary Table 3. Dimensions of the warp and weft crystals in the crystalline patches **A**1, **A**2, **B**, **C**1, **C**2 and **C**3

Patch	Yarn	Dimension / µm	Crystal numbers				
			1	2	3	4	5
**A**1	Wefts	Width	99.68	108.50	82.91	105.02	91.00
		Thickness	10.68	11.32	9.64	11.20	9.73
	Warps	Width	89.02	93.45	114.62	131.65	126.80
		Thickness	9.24	10.62	12.01	14.09	15.79
**A**2	Wefts	Width	239.64	218.86	177.08	197.59	198.47
		Thickness	25.74	21.03	16.08	27.96	22.29
	Warps	Width	156.23	166.77	170.89	187.88	237.12
		Thickness	18.11	21.12	32.63	24.97	26.23
**B**	Wefts	Width	81.18	59.95	94.14	46.15	47.86
		Thickness	39.50	23.53	29.55	31.04	22.19
	Warps	Width	104.91	74.37	81.69	76.41	91.27
		Thickness	40.62	32.82	42.63	28.96	38.80
**C**1	Wefts	Width	93.74	122.03	91.82	102.91	58.94
		Thickness	36.33	47.11	46.84	53.92	26.47
	Warps	Width	141.50	110.32	98.53	111.48	93.03
		Thickness	66.73	42.42	48.41	56.18	34.54
**C**2	Wefts	Width	286.39	207.41	187.78	296.12	258.50
		Thickness	52.07	53.18	64.76	61.74	56.20
	Warps	Width	266.53	357.27	262.51	265.49	245.59
		Thickness	66.73	94.57	79.84	44.86	40.21
**C**3	Wefts	Width	127.28	132.02	131.83	122.87	138.90
		Thickness	23.03	33.71	28.94	19.81	24.22
	Warps	Width	175.50	156.32	138.53	149.48	136.03
		Thickness	66.73	42.42	48.41	56.18	34.54

Supplementary Table 4. Dimensions of the warp and weft crystals in the crystalline patches **A#B**, **A#C**, **B#C**, **A#B#C** and **D** (twisted)

Patch	Yarn	Dimension / μm	Crystal numbers				
			1	2	3	4	5
**A**#B	Wefts	Width	100.70	117.31	103.64	99.27	110.55
		Thickness	11.41	40.44	10.46	31.09	9.07
	Warps	Width	134.84	80.31	106.59	76.72	101.78
		Thickness	15.13	37.71	12.56	39.35	12.88
**A**#C	Wefts	Width	190.58	180.89	195.83	208.46	159.05
		Thickness	21.57	32.26	22.40	48.30	10.98
	Warps	Width	167.11	159.48	178.92	238.67	204.07
		Thickness	20.35	24.21	15.77	57.32	20.38
**B#C**	Wefts	Width	60.70	80.31	38.06	76.72	54.06
		Thickness	34.10	21.25	18.79	44.41	27.16
	Warps	Width	79.08	117.29	48.47	99.27	69.32
		Thickness	30.62	36.66	23.08	40.35	40.42
**A#B#C**	Wefts	Width	89.30	61.54	126.3	97.26	37.49
		Thickness	9.89	30.17	32.58	10.18	21.06
	Warps	Width	107.90	66.55	94.36	106.4	68.06
		Thickness	10.85	24.29	45.81	14.43	22.54
**D** (twisted)	Wefts	Width	88.52	69.48	50.63	69.72	41.67
		Thickness	82.78	61.36	43.34	65.49	38.25
	Warps	Width	53.98	86.92	62.85	89.78	65.83
		Thickness	46.53	83.44	57.66	76.82	54.40

Supplementary Table 5. Comparison of the dimensions of the woven crystals in crystalline patches and the woven structures[1–7] obtained by other methods such as electrospinning

Woven structures	Dimensions (width or diameter) / μm	Standard deviation / μm
**A**1	104.26	16.28
**A**2	195.05	29.07
**B**	75.79	19.44
**C**1	102.43	21.70
**C**2	263.36	46.69
**C**3	140.88	15.68
**A**#**B**	103.12	17.73
**A**#C	188.31	24.79
**B**#**C**	72.33	23.80
**A**#**B**#**C**	85.52	26.62
**D** (twisted)	67.94	16.60
PVP (doped-crystals)[1]	2	—
PVDF fibers[2]	40–120	—
PAN nanofibers[3]<br>Viscose composite yarns[3]	0.28–0.84<br>11.57	—
PAN fibers[4]	0.35–0.50	—
GelMA fibers[5]<br>Crosslinked gelatin fibers[5]	2.18<br>1.76	0.52<br>0.45
PCL NY[6]<br>PCL/SF-4/1 NY[6]<br>PCL/SF-3/2 NY[6]	0.4533<br>0.6133<br>0.9508	0.0553<br>0.0488<br>0.0898
RP-PCL[7]<br>EHD-PCL[7]	220<br>205	25<br>61

Supplementary Table 6. Average crystal size and structural parameters of eleven crystalline patches

Patch	Yarn	Average Yarn Width / μm	Average Thickness / μm	Average grid size / mm	Patch width and length / mm	Threads per unitlength (10 cm)	Patch tightness / %	Covering factor / %	Weight / mg	Bulk density / g cm^{-3}
**A**1	Warp	111.11	12.35	0.41	2.12	189	21.04	39.05	0.16	2.50
	Weft	97.42	10.51	0.52	2.64	236	22.98			
**A**2	Warp	183.78	24.61	0.74	4.01	119	21.88	42.35	0.24	0.60
	Weft	206.33	22.62	0.82	4.20	125	25.73			
**B**	Warp	85.73	36.77	0.95	4.13	77	6.59	13.94	0.36	0.41
	Weft	65.86	29.16	1.52	6.50	121	7.97			
**C**1	Warp	110.97	49.66	1.61	6.90	67	7.42	13.65	1.40	0.59
	Weft	93.89	42.13	1.73	7.48	72	6.80			
**C**2	Warp	279.48	65.24	1.78	8.35	57	16.06	28.26	3.92	0.87
	Weft	247.24	57.59	1.83	8.70	60	14.80			
**C**3	Warp	151.16	31.01	0.99	4.60	98	14.86	26.79	1.20	1.80
	Weft	130.58	25.94	1.08	5.08	109	14.19			
**A#B**	Warp	97.37	25.05	0.70	4.10	101	9.80	22.66	0.52	0.88
	Weft	99.63	21.72	1.08	5.96	146	14.58			
**A#C**	Warp	195.60	30.93	1.18	7.01	71	13.94	27.53	0.93	0.52
	Weft	184.71	29.78	1.45	8.42	86	15.81			
**B#C**	Warp	79.96	33.92	1.36	7.19	78	6.20	11.11	0.56	0.31
	Weft	65.64	31.12	1.45	7.74	83	5.48			
**A#B#C**	Warp	93.36	29.53	0.73	4.20	133	12.45	23.72	0.71	1.40
	Weft	87.46	23.40	0.79	4.50	142	12.49			
**D** (twisted)	Warp	71.87	63.77	1.36	5.75	76	5.47	10.58	0.57	0.25
	Weft	64.00	58.24	1.55	6.56	87	5.56			

Supplementary Table 7. Dimensions of crystals **A** and **C** in crystalline patches with different topologies (plain, twill and stain)

Wefts (**A**)	Width / μm	615.38	486.80	434.62	342.58	435.26	332.10	377.53	451.04	518.13	563.48
	Thickness / μm	62.10	31.59	35.85	46.32	33.45	29.66	29.26	31.45	42.82	38.55
Warps (**C**)	Width / μm	422.69	521.55	544.68	465.49	352.30	485.65	486.09	480.85	493.34	382.55
	Thickness / μm	57.55	76.54	57.38	62.30	60.45	73.75	51.74	52.17	38.48	43.24

Supplementary Table 8. Average crystal size and structural parameters of crystalline patches with different structures including plain, twill and stain

<table>
<tr><th>Patch</th><th>Yarn</th><th>Average Yarn Width / μm</th><th>Average Thickness / μm</th><th>Average grid size / mm</th><th>Patch width and length / mm</th><th>Threads per unitlength (10 cm)</th><th>Patch tightness / %</th><th>Covering factor / %</th><th>Weight / mg</th><th>Bulk density / g cm^{-3}</th></tr>
<tr><td rowspan="2">**A1**</td><td rowspan="6">Warp
Weft</td><td rowspan="6">463.52
455.69</td><td rowspan="6">57.36
38.10</td><td>1.66</td><td>19.50</td><td>42</td><td>19.31</td><td rowspan="2">33.73</td><td rowspan="2">0.16</td><td rowspan="2">2.50</td></tr>
<tr><td>2.15</td><td>24.00</td><td>51</td><td>23.36</td></tr>
<tr><td rowspan="2">**A2**</td><td>0.87</td><td>11.56</td><td>69</td><td>31.84</td><td rowspan="2">42.35</td><td rowspan="2">0.24</td><td rowspan="2">0.60</td></tr>
<tr><td>1.10</td><td>13.87</td><td>81</td><td>36.81</td></tr>
<tr><td rowspan="2">**B**</td><td>0.28</td><td>7.05</td><td>117</td><td>54.40</td><td rowspan="2">13.94</td><td rowspan="2">0.36</td><td rowspan="2">0.41</td></tr>
<tr><td>0.43</td><td>8.52</td><td>142</td><td>64.64</td></tr>
</table>

Supplementary Table 9. Average crystal size and structural parameters of crystalline patches **C4** and **C5**

<table>
<tr><th>Patch</th><th>Yarn</th><th>Average Yarn Width / μm</th><th>Average Thickness / μm</th><th>Average grid size / mm</th><th>Patch width and length / mm</th><th>Threads per unitlength (10 cm)</th><th>Patch tightness / %</th><th>Covering factor / %</th><th>Weight / mg</th><th>Bulk density / g cm^{-3}</th></tr>
<tr><td rowspan="2">**C4**</td><td>Warp</td><td>558.67</td><td>89.56</td><td>0.26</td><td>21.93</td><td>123</td><td>68.78</td><td rowspan="2">73.21</td><td rowspan="2">432</td><td rowspan="2">10.81</td></tr>
<tr><td>Weft</td><td>543.23</td><td>82.04</td><td>4.52</td><td>20.80</td><td>24</td><td>13.06</td></tr>
<tr><td rowspan="2">**C5**</td><td>Warp</td><td>187.39</td><td>31.94</td><td>0.07</td><td>22.78</td><td>394</td><td>73.82</td><td rowspan="2">74.76</td><td rowspan="2">326</td><td rowspan="2">32.25</td></tr>
<tr><td>Weft</td><td>214.87</td><td>42.23</td><td>7.31</td><td>11.93</td><td>17</td><td>3.77</td></tr>
</table>

Supplementary Table 10. The distance between the cross nodes of warp and weft of the patch **A#C** and the output terminals of L, U, R and D directions.

Wefts (L&R) \ Warps (U&D)		1 (12.2 mm)	2 (12.2 mm)	3 (12.2 mm)	4 (12.4 mm)	5 (12.5 mm)	6(12.6 mm)
1 (12.5 mm)	L / mm	3.5	4.7	5.8	6.7	7.7	9.1
	U / mm	3.1	3.6	3.7	3.8	4.0	3.6
	R / mm	9.0	7.8	6.7	5.8	4.8	3.4
	D / mm	9.1	8.6	8.5	8.6	8.5	9.0
2 (12.5 mm)	L / mm	3.5	4.7	5.8	6.7	7.8	9.0
	U / mm	4.2	4.6	4.8	4.8	5.0	4.6
	R / mm	9.0	7.8	6.7	5.8	4.7	3.5
	D / mm	8.0	7.6	7.4	7.6	7.5	8.0
3 (12.5 mm)	L / mm	3.5	4.8	5.9	6.9	7.9	9.1
	U / mm	5.4	5.7	5.9	5.9	6.2	5.5
	R / mm	9.0	7.7	6.6	5.5	4.6	3.4
	D / mm	6.8	6.5	6.3	6.5	6.3	7.1
4 (12.5 mm)	L / mm	3.6	5.0	6.0	7.0	8.0	9.3
	U / mm	6.2	6.6	6.9	6.9	7.0	6.7
	R / mm	8.9	7.5	6.5	5.5	4.5	3.2
	D / mm	6.0	5.6	5.3	5.5	5.5	5.9
5 (12.6 mm)	L / mm	3.7	5.0	6.1	7.1	8.1	9.4
	U / mm	7.7	8.1	8.5	8.3	8.5	8.1
	R / mm	8.9	7.6	6.5	5.5	4.5	3.2
	D / mm	4.5	4.1	3.7	4.1	4.0	4.5
6 (12.7 mm)	L / mm	3.4	4.6	5.6	6.8	7.8	9.0
	U / mm	9.2	9.5	9.8	9.8	9.9	9.5
	R / mm	9.3	8.1	7.1	5.9	4.9	3.7
	D / mm	3.0	2.7	2.4	2.6	2.6	3.1

Supplementary Table 11. The distance between the cross nodes of warp and weft of the patch **A#B#C** and the output terminals of L, U, R and D directions.

Wefts (L&R) \ Warps (U&D)		1 (10.5 mm)	2 (10.4 mm)	3 (10.4 mm)	4 (10.4 mm)	5 (10.3 mm)	6(10.4 mm)
1 (10.0 mm)	L / mm	2.8	3.7	4.6	5.7	6.6	7.4
	U / mm	3.0	3.0	3.0	3.0	3.0	3.0
	R / mm	8.2	6.3	5.4	4.3	3.4	2.6
	D / mm	7.5	7.4	7.4	7.4	7.3	7.4
2 (9.7 mm)	L / mm	2.6	3.5	4.4	5.5	6.4	7.2
	U / mm	4.0	4.0	4.0	3.8	3.8	3.8
	R / mm	7.1	6.2	5.3	4.2	3.3	2.5
	D / mm	6.5	6.4	6.4	6.6	6.5	6.6
3 (9.8 mm)	L / mm	2.8	3.6	4.6	5.6	6.4	7.3
	U / mm	4.7	4.7	4.7	4.6	4.7	4.7
	R / mm	7.0	6.2	5.2	4.2	3.4	2.5
	D / mm	5.8	5.7	5.7	5.8	5.7	5.7
4 (9.9 mm)	L / mm	2.7	3.6	4.4	5.5	6.3	7.2
	U / mm	6.0	5.9	5.9	5.9	5.9	5.9
	R / mm	7.2	6.3	5.5	4.4	3.6	2.7
	D / mm	4.5	4.5	4.5	4.5	4.4	4.5
5 (10.0 mm)	L / mm	2.7	3.5	4.4	5.4	6.2	7.1
	U / mm	7.1	7.0	7.0	7.0	7.0	7.0
	R / mm	7.3	6.5	5.6	4.6	3.8	2.9
	D / mm	3.3	3.4	3.4	3.4	3.3	3.4
6 (10.0 mm)	L / mm	2.7	3.6	4.4	5.4	6.2	7.1
	U / mm	7.9	7.4	7.8	7.7	7.7	7.7
	R / mm	7.3	6.4	5.6	4.6	3.8	2.9
	D / mm	2.6	3.0	2.6	2.7	2.6	2.7

4. Supplementary references

1. T. Wang, Y. Song, M. Liu, C. Gao, H. Yang, L. Wang, D. Liu, T. Wang, W. Hu, Electrospinning enables flexibility of organic long-persistent luminescence crystals, *Dyes Pigm.* **2020**, *207*, 110734.
2. X. Meng, Q. Li, Z. Hu, M. Guo, Microfluidic fabrication of β-phase enriched poly(vinylidene fluoride) microfibers toward flexible piezoelectric sensor, *J Polym. Sci.* **2022**, *60*, 1718–1726.
3. Y. Yang, Y. Zhao, Z. Quan, H. Zhang, X. Qin, R. Wang, J. Yu, An efficient hybrid strategy for composite yarns of micro-/nano-fibers, *Mater. Design* **2019**, *184,* 108196.
4. X. Yin, Y. Zhang, X. Xu, Y. Wang, Bilayer fiber membrane electrospun from MOF derived Co_3S_4 and PAN for solar steam generation induced sea water desalination, *J Solid State Chem.* **2021**, *303* 122423.
5. X. Sun, Q. Lang, H. Zhang, L. Cheng, Y. Zhang, G. Pan, X. Zhao, H. Yang, Y. Zhang, H. A. Santos, W. Cui, Electrospun photocrosslinkable hydrogel fibrous scaffolds for rapid in vivo vascularized skin flap regeneration, *Adv. Funct. Mater.* **2017**, *27*, 1604617.
6. Y. Qi, C. Wang, Q. Wang, F. Zhou, T. Li, B. Wang, W. Su, D. Shang, S. Wu, A simple, quick, and cost-effective strategy to fabricate polycaprolactone/silk fibroin nanofiber yarns for biotextile-based tissue scaffold application, *Eur. Polym. J* **2023**, *186*, 111863.
7. S. H. Ahn, H. J. Lee, G. H. Kim, Polycaprolactone scaffolds fabricated with an advanced electrohydrodynamic direct-printing method for bone tissue regeneration, *Biomacromolecules* **2011**, *12*, 4256–4263.
