# Supplementary material for: Woven organic crystals
Source: Nat Commun. 2023 Nov 21;14:7582. doi: 10.1038/s41467-023-43084-7 (PMC10663483; doi:10.1038/s41467-023-43084-7)
Supplement: Supplementary file 3 — Description of Additional Supplementary Files [file 41467_2023_43084_MOESM3_ESM.pdf]

<u>Description of additional supplementary files</u>

Supplementary Movie 1. Comparison of the stability of different forms of fixed crystalline patches, including unfixed, fixed nodes at four corners, and fixed nodes along the perimeter.

Supplementary Movie 2. The three-point bending of single crystals of **A** and **C**, and three-point bending of crystalline patches from **A** and **C** along different directions.

Supplementary Movie 3. Repeated bending of patches **B**, **A** and **C** at room temperature (RT) and immersed in liquid nitrogen (LN), respectively. The patches were attached to a black paper which was subsequently bent to bend the patch.
